# Reclaiming saliency: Rhythmic precision-modulated action and perception

**DOI:** 10.3389/fnbot.2022.896229

**Published:** 2022-07-28

**Authors:** Ajith Anil Meera, Filip Novicky, Thomas Parr, Karl Friston, Pablo Lanillos, Noor Sajid

**Affiliations:** ^1^Department of Cognitive Robotics, Faculty of Mechanical, Maritime and Materials Engineering, Delft University of Technology, Delft, Netherlands; ^2^Department of Neurophysiology, Donders Institute for Brain Cognition and Behavior, Radboud University, Nijmegen, Netherlands; ^3^Wellcome Centre for Human Neuroimaging, University College London, London, United Kingdom; ^4^Department of Artificial Intelligence, Donders Institute for Brain Cognition and Behavior, Radboud University, Nijmegen, Netherlands

**Keywords:** attention, saliency, free-energy principle, active inference, precision, brain-inspired robotics, cognitive robotics

## Abstract

Computational models of visual attention in artificial intelligence and robotics have been inspired by the concept of a saliency map. These models account for the mutual information between the (current) visual information and its estimated causes. However, they fail to consider the circular causality between perception and action. In other words, they do not consider where to sample next, given current beliefs. Here, we reclaim salience as an active inference process that relies on two basic principles: uncertainty minimization and rhythmic scheduling. For this, we make a distinction between attention and salience. Briefly, we associate attention with precision control, i.e., the confidence with which beliefs can be updated given sampled sensory data, and salience with uncertainty minimization that underwrites the selection of future sensory data. Using this, we propose a new account of attention based on rhythmic precision-modulation and discuss its potential in robotics, providing numerical experiments that showcase its advantages for state and noise estimation, system identification and action selection for informative path planning.

## 1. Introduction

Attention is a fundamental cognitive ability that determines which events from the environment, and the body, are preferentially processed (Itti and Koch, 2001). For example, the motor system directs the visual sensory stream by orienting the fovea centralis (i.e., the retinal region of highest visual acuity) toward points of interest within the visual scene. Thus, the confidence with which the causes of sampled visual information are inferred is constrained by the physical structure of the eye—and eye movements are necessary to minimize uncertainty about visual percepts (Ahnelt, 1998). In neuroscience, this can be attributed to two distinct, but highly interdependent attentional processes: (*i*) attentional gain mechanisms reliant on estimating the sensory precision of current data (Feldman and Friston, 2010; Yang et al., 2016a), and (*ii*) attentional salience that involves actively engaging with the sensorium to sample appropriate future data (Lengyel et al., 2016; Parr and Friston, 2019). Here we refer to perceptual-related salience, i.e., processing of low-level visual information (Santangelo, 2015). Put simply, we formalize the fundamental difference between attention—as optimizing perceptual processing—and salience as optimizing the sampling of what is processed. This highlights the dynamic, circular nature with which biological agents acquire, and process, sensory information.

Understanding the computational mechanisms that undergird these two attentional phenomena is pertinent for deploying apt models of (visual) perception in artificial agents (Klink et al., 2014; Mousavi et al., 2016; Atrey et al., 2019) and robots (Frintrop and Jensfelt, 2008; Begum and Karray, 2010; Ferreira and Dias, 2014; Lanillos et al., 2015a). Previous computational models of visual attention, used in artificial intelligence and robotics, have been inspired (and limited) by the feature integration theory proposed by Treisman and Gelade (1980) and the concept of a saliency map (Tsotsos et al., 1995; Itti and Koch, 2001; Borji and Itti, 2012). Briefly, a saliency map is a static two-dimensional ‘image' that encodes stimulus relevance, e.g., the importance of particular region. These maps are then used to isolate relevant information for control (e.g., to direct foveation of the maximum valued region). Accordingly, computational models reliant on this formulation do not consider the circular-dependence between action selection and cue relevance—and simply use these static saliency maps to guide action.

In this article, we adopt a first principles account to disambiguate the computational mechanisms that underpin attention and salience (Parr and Friston, 2019) and provide a new account of attention. Specifically, our formulation can be effectively implemented for robotic systems and facilitates both state-estimation and action selection. For this, we associate attention with precision control, i.e., the confidence with which beliefs can be updated given (current) sampled sensory data. Salience is associated with uncertainty minimization that influences the selection of future sensory data. This formulation speaks to a computational distinction between action selection (i.e., where to look next) and visual sampling (i.e., what information is being processed). Importantly, recent evidence demonstrates the rhythmic nature of these processes *via* a theta-cycle coupling that fluctuates between high and low precision—as unpacked in Section 2. From a robotics perspective, resolving uncertainty about states of affair speaks to a form of Bayesian optimality, in which decisions are made to maximize expected information gain (Lindley, 1956; Friston et al., 2021; Sajid et al., 2021a). The duality between attention and salience is important for resolving uncertainty and enabling active perception. Significantly, it addresses an important challenge for defining autonomous robotics systems that can balance optimally between data assimilation (i.e., confidently perceiving current observations) and exploratory behavior to maximize information gain (Bajcsy et al., 2018).

In what follows, we review the neuroscience of attention and salience (Section 2) to develop a novel (computational) account of attention based on precision-modulation that underwrites perception and action (Section 3). Next, we face-validate our formulation within a robotics context using numerical experiments (Section 4). The robotics implementation instantiates a free energy principle (FEP) approach to information processing (Friston, 2010). This allows us to modulate the (appropriate) precision parameters to solve relevant robotics challenges in perception and control; namely, state-estimation (Section 4.2.2), system identification (Section 4.2.3), planning (Section 4.3), and active perception (Section 4.3.3). We conclude with a discussion of the requisite steps for instantiating a full-fledged computational model of precision-modulated attention—and its implications in a robotics setting.

## 2. Attention and salience in neuroscience

Our interactions with the world are guided by efficient gathering and processing of sensory information. The quality of these acquired sensory data is reflected in attentional resources that select sensations which influence our beliefs about the (current and future) states of affairs (Lengyel et al., 2016; Yang et al., 2016b). This selection is often related to gain control, i.e., an increase of neural spikes when an object is attended to. However, gain control only accounts for half the story because we can only attend to those objects that are within our visual field. Accordingly, if a salient object is outside the center of our visual field, we orient the fovea to points of interest. This involves two separate, but often conflated, processes: attention and salience—where the former relates to processing current visual data, and the latter to ensuring the agent samples salient data in the future (Parr and Friston, 2019). That these two processes are strongly coupled is exemplified by the pre-motor theory of attention (Rizzolatti et al., 1987), which highlights the close relationship between overt saccadic sampling of the visual field and the covert deployment of attention in the absence of eye movements. Specifically, it posits that covert attention^1^ is realized *via* processes that are generated by particular eye movements but inhibits the action itself. In this sense, it does not distinguish between covert and overt^2^ types of attention.

From a first principles (Bayesian) account, it is necessary to separate between attention and salience because they speak to different optimization processes. Explicitly, attention as a precision-dependent (neural) gain control mechanism that facilitates optimization of the *current* sampled sensory data (Desimone, 1996; Feldman and Friston, 2010). Conversely, salience is associated with selection of *future* data that reduces uncertainty (Friston et al., 2015; Mirza et al., 2016; Parr and Friston, 2019). Put simply, it is possible to optimize attention in the absence of eye movements and active vision, whereas salience is necessary to optimize the deployment of eye movements. In what follows, we formalize this distinction with a particular focus on visual attention (Kanwisher and Wojciulik, 2000), and discuss recent findings that speak to a rhythmic coupling that underwrites periodic deployment of gain control and saccades, *via* modulation of distinct precision parameters.

### 2.1. Attention as neural gain control

Neural gain control can be regarded as an amplifier of neural communication during attention tasks (Reynolds et al., 2000; Eldar et al., 2013). Computationally, this is analogous to modulating a precision term, or the inverse temperature parameter (Feldman and Friston, 2010; Parr and Friston, 2017a). For this reason, we refer to precision and gain control interchangeably. An increase in gain amplifies the postsynaptic responses of neurons to their pre-synaptic input. Thus, gain control rests on synaptic modulation that can emphasize—or preferentially select—a particular type of sensory data. From a Bayesian perspective (Rao, 2005; Spratling, 2008; Parr et al., 2018), this speaks to the confidence with which beliefs can be updated given sampled sensory data (i.e., optimal state estimation)—under a generative model (Whiteley and Sahani, 2008; Parr et al., 2018). For example, affording high precision to certain sensory inputs would lead to confident Bayesian belief updating. However, low precision reduces the influence of sensory input by attenuating the precision of the likelihood, relative to a prior belief, and current observations would do little to resolve ensuing uncertainty. Thus, sampled visual data (from different areas) can be predicted with varying levels of precision, where attention accentuates sensory precision. The deployment of precision or attention is influenced by competition between stimuli (i.e., which sensory data to sample) and prior beliefs. Interestingly, casting attention as precision or, equivalently, synaptic gain offers a coherency between biased competition (Desimone, 1996), predictive coding (Spratling, 2008) and generic active inference schemes (Feldman and Friston, 2010; Brown et al., 2013; Kanai et al., 2015; Parr et al., 2018).

Naturally, gain control is accompanied by neuronal variability, i.e., sharpened neural responses for the same task over time. Consistent with gain control, these fluctuations in neural responses across trials can be explained by precision engineered message passing (Clark, 2013) *via* (*i*) normalization models (Reynolds and Heeger, 2009; Ruff and Cohen, 2016), (*ii*) temperature parameter manipulation (Feldman and Friston, 2010; Parr and Friston, 2017a; Parr et al., 2018, 2019; Mirza et al., 2019), or (*iii*) introduction of (conjugate hyper-)priors that are either pre-specified (Sajid et al., 2020, 2021b) or optimized using uninformed priors (Friston et al., 2003; Anil Meera and Wisse, 2021). Recently, these approaches have been used to simulate attention by accentuating predictions about a given visual stimulus (Reynolds and Heeger, 2009; Feldman and Friston, 2010; Ruff and Cohen, 2016). For example, normalization models propose that every neuronal response is normalized within its neuronal ensemble (i.e., the surrounding neuronal responses) (Heeger, 1992; Louie and Glimcher, 2019). Thus, to amplify the neuronal response of particular neuron, the neuronal pool has to be inhibited such that particular neuron has a sharper evoked response (Schmitz and Duncan, 2018). Importantly, these (superficially distinct) formulations simulate similar functions using different procedures to accentuate responses over a particular neuronal pool for a given neuron or a group of neurons. This introduces shifts in precision to produce attentional gain and the precision of neuronal encoding.

### 2.2. Salience as uncertainty minimization

In the neurosciences, (visual) salience refers to the ‘significance' of particular objects in the environment. Salience often implicates the superior colliculus, a region that encodes eye movements (White et al., 2017). This makes intuitive sense, as the superior colliculus plays a role in generation of eye movements—being an integral part of the brainstem oculomotor network (Raybourn and Keller, 1977)—and salient objects provide information that is best resolved in the center of the visual field, thus motivating eye movements to that location. For this reason, our understanding of salience is a quintessentially action-driving phenomenon (Parr and Friston, 2019). Mathematically, salience has been defined as Bayesian surprise (Itti and Koch, 2001; Itti and Baldi, 2009), intrinsic motivation (Oudeyer and Kaplan, 2009), and subsequently, epistemic value under active inference (Mirza et al., 2016; Parr et al., 2018). Active inference—a Bayesian account of perception and action (Friston et al., 2017a; Da Costa et al., 2020)—stipulates that action selection is determined by uncertainty minimization. Formally, uncertainty minimization speaks to minimization of an expected free energy functional over future trajectories (Da Costa et al., 2020; Sajid et al., 2021a). This action selection objective can be decomposed into epistemic and extrinsic value, where the former pertains to exploratory drives that encourage resolution of uncertainty by sampling salient observations, e.g., only checking one's watch when one does not know the time. However, after checking the watch there is little epistemic value in looking at it again. Generally, the tendency to seek out new locations—once uncertainty has been resolved at the current fixation point—is called inhibition of return (Klein, 2000).

From an active inference perspective, this phenomenon is prevalent because a recent action has already resolved the uncertainty about the time and checking again would offer nothing more in terms of information gain (Parr and Friston, 2019). Accordingly, salience involves seeking sensory data that have a predictable, uncertainty reducing, effect on current beliefs about states of affairs in the world (Mirza et al., 2016; Parr et al., 2018). Thus salience contends with beliefs about data that must be acquired and the precision of beliefs about policies (i.e., action trajectories) that dictate it. Formally, this emerges from the imperative to maximize the amount of information gained regarding beliefs, from observing the environment. Happily, prior studies have made the connection between eye movements, salience, and precision manipulation (Friston et al., 2011; Brown et al., 2013; Crevecoeur and Kording, 2017). This connection emerges from planning strategies that allow the agent to minimize uncertainty by garnering the right kind of data.

Next, we consider recent findings on how the coupling of these two mechanisms, attention and salience, may be realized in the brain.

### 2.3. Rhythmic coupling of attention and salience

To illustrate the coupling between attention and salience, we turn to a recent rhythmic theory of attention. The theory proposes that coupling of saccades, during sampling of visual information, happens at neuronal and behavioral theta oscillations; a frequency of 3–8 Hz (Fiebelkorn and Kastner, 2019, 2021). This frequency simultaneously allows for: (*i*) a systematic integration of visual samples with action, and (*ii*) a temporal schedule to disengage and search the environment for more relevant information.

Given that gain control is related to increased sensory precision, we can accordingly relate saccadic eye movements to the decreased precision. This introduces saccadic suppression, a phenomenon that decreases visual gain during eye movements (Crevecoeur and Kording, 2017). This phenomenon was described by Helmholtz who observed that externally initiated eye movements (e.g., when oneself gently presses a side of an eye) eludes the saccadic suppression that accompanies normal eye movements—and we see the world shift, because optic flow is not attenuated (Helmholtz, 1925). An interesting consequence of this is that, as eye movements happen periodically (Rucci et al., 2018; Benedetto et al., 2020), there must be a periodic switch between high and low sensory precision, with high precision (or enhanced gain) during fixations and low precision (or suppressed gain) during saccades. Interestingly, it has been shown that rather than having action resetting the neural periodicity, it is better understood as something that aligns within an already existing rhythm (Hogendoorn, 2016; Tomassini et al., 2017). Additionally, the rhythmicity of higher and lower fidelity of sensory sampling has been shown to fluctuate rhythmically around 3 Hz (Benedetto and Morrone, 2017), suggesting that action emerges rhythmically when visual precision is low (Hogendoorn, 2016), triggering salience.

Building upon this, we hypothesize that theta rhythms generated in the fronto-parietal network (Fiebelkorn et al., 2018; Helfrich et al., 2018; Fiebelkorn and Kastner, 2020) couples saccades with saccadic suppression causing the switches between visual sampling and saccadic shifting. This introduces a diachronic aspect to the belief updating process (Friston et al., 2020; Parr and Pezzulo, 2021; Sajid et al., 2022); i.e., sequential fluctuations between attending to current data (perception) and seeking new data (action). This supports empirical findings that both eye movements (Sommer and Wurtz, 2006) and filtering irrelevant information (Phillips et al., 2016; Nakajima et al., 2019; Fiebelkorn and Kastner, 2020) are initiated in this cortical network. Interestingly, both eye movements and visual filtering then propagate to sub-cortical regions, i.e., the superior colliculus—for saliency map composition (White et al., 2017)—and the thalamus—for gain control (Kanai et al., 2015; Fiebelkorn et al., 2019), respectively. Furthermore, this is consistent with recent findings that the periodicity of neural responses are important for understanding the relation of motor responses and sensory information—i.e., perception-action coupling (Benedetto et al., 2020). Importantly, theta rhythms also speak to the speed (i.e., the temporal schedule) with which visual information is sampled from the environment (Busch and VanRullen, 2010; Dugué et al., 2015, 2016; Helfrich et al., 2018). Meaning visual information is not sampled continuously, as our visual experiences would suggest, but rather it is made of successive discrete samples (VanRullen, 2016; Parr et al., 2021).

The prefrontal theta rhythm has been associated with working memory (WM), a process that holds compressed information about the previously observed stimuli, in the sense that measured power in this frequency range using electroencephalography increases during tasks that place demands on WM (Axmacher et al., 2010; Hsieh and Ranganath, 2014; Köster et al., 2018; Brzezicka et al., 2019; Peters et al., 2020; Balestrieri et al., 2021; Pomper and Ansorge, 2021). The implication is that the neural processes that underwrite WM may depend upon temporal cycles with periods similar to that of perceptual sampling. Importantly, this cognitive process is influenced by how salient a particular stimulus was (Fine and Minnery, 2009; Santangelo and Macaluso, 2013; Santangelo et al., 2015). Moreover, WM has been implicated with attentional mechanisms (Knudsen, 2007; Gazzaley and Nobre, 2012; Oberauer, 2019; Peters et al., 2020; Panichello and Buschman, 2021). This is aligned with our account where we illustrate a rhythmic coupling between salience and attention.

In summary, the computations that underwrite attention and active vision are coupled and exhibit circular causality. Briefly, selective attention and sensory attenuation optimize the processing of sensory samples and which particular visual percepts are inferred. In turn, this determines appropriateness of future eye movements (or actions) and shapes which prior stimuli are encoded into the agent's working memory. Interestingly, the close functional (and computational) link between the two mechanisms endorses the pre-motor theory of attention.

## 3. Proposed precision-modulated account of attention and salience

**Figure 1:**
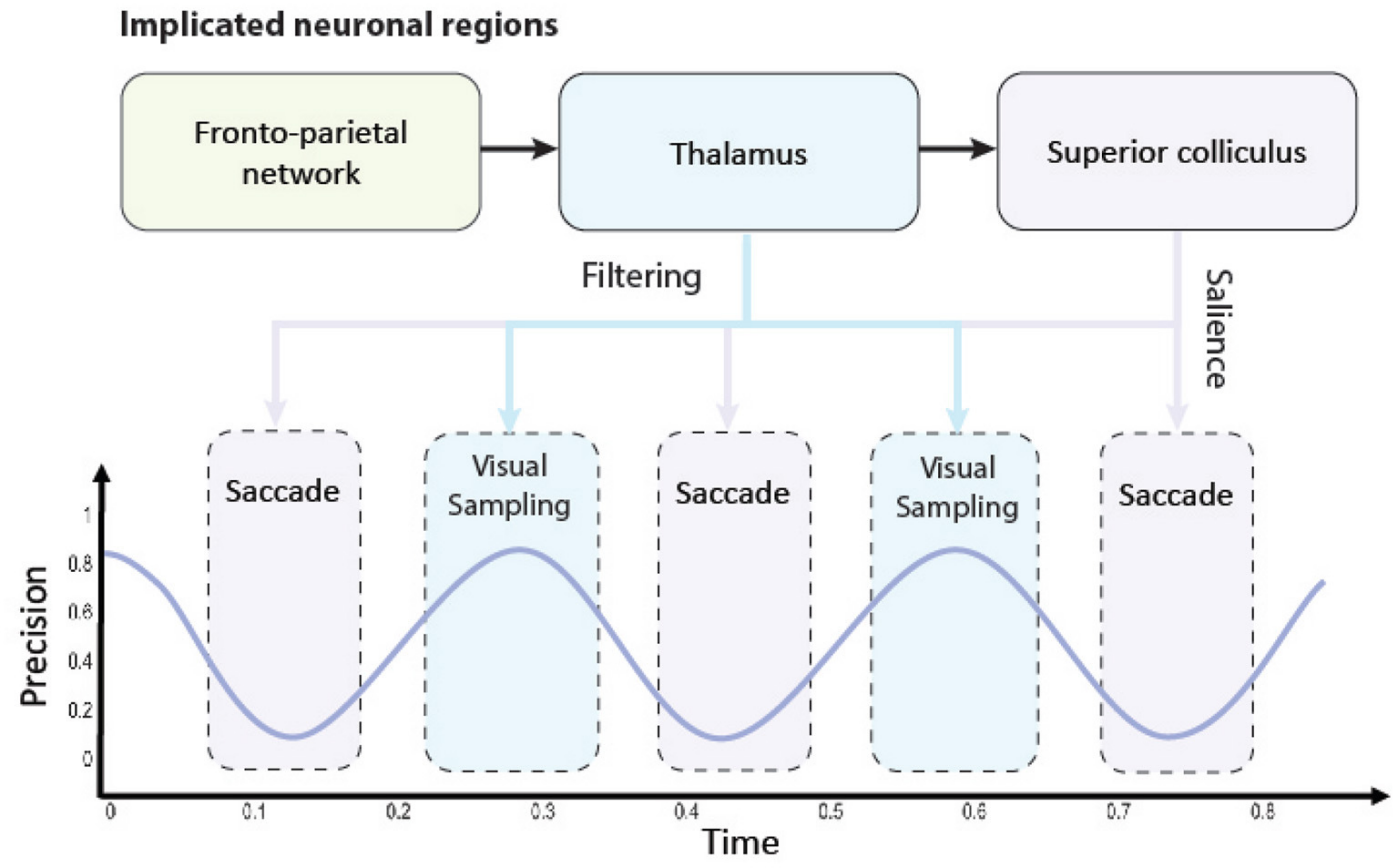
A graphical illustration of the precision-modulated account of perception and action. Salience and attention are computed based upon beliefs (assumed to be) encoded in parts of the fronto-parietal network and realized in distinct brain regions: superior colliculus (SC) for perception as inference and thalamus for planning as inference, respectively. To deploy attentional processes efficiently, these two mechanisms have to be aligned, which is done rhythmically, hypothetically in theta frequency. This coupling enables the saccadic suppression phenomenon through fluctuations in precision (on an arbitrary scale). When precision is low (i.e., the trough of the theta rhythm), the saccade emerges. Note that there might be distinct processes inhibiting the action (e.g., covert attention), and (despite a decline in precision) saccades might not emerge in every theta cycle. On the other hand, high precision facilitates confident inferences about the causes of visual data. Under this account, thalamus is used for initiating gain control (or visual sampling in general) by providing stronger sensory input, while superior colliculus dictates next saccades, that lead to most informative fixation positions.

Here, we introduce our precision-modulated account of perception and action. A graphical illustration is provided in Figure 1. For this, we turn to attention and salient action selection which have their roots in biological processes relevant for acquiring task-relevant information. Under an active inference account, this attention influences (posterior) state estimation and can be associated with increased precision of belief updating and gain control—described in Section 2.1. Furthermore, this is distinct from salience despite interdependent neuronal composition and computations.

Further alignment between the two constructs can be revealed by considering the temporal scheduling between movement (i.e., action) and perception for uncertainty resolution (Parr and Friston, 2019). We postulate that this perception-action coupling is best understood as a periodic fluctuation between minimizing uncertainty and precision control. Subsequently, action is deployed to reduce uncertainty. Such an alignment specifies what stimulus is selected and under what level of precision it is processed. Parr and Friston (2019) hypothesize that action alignment with precision is due to the eye structure that provides precise information in the fovea and requires the agent to foveate the most informative stimulus. We extend this by considering the periodic deployment of gain control with saccades (Hogendoorn, 2016; Benedetto and Morrone, 2017; Tomassini et al., 2017; Fiebelkorn and Kastner, 2019; Nakayama and Motoyoshi, 2019).

Accordingly, our formulation defines attention as precision control and salience as uncertainty minimization supported by discrete sampling of visual information at a theta rhythm. This synchronizes perception and action together in an oscillatory fashion (Hogendoorn, 2016). Importantly, a Bayesian formulation of this can be realized as precision manipulation over particular model parameters. We reserve further details for Section 4.

**Summary** Based upon our review, we propose a precision-modulated account of attention and salience, emphasizing the diachronic realization of action and perception. In the following sections, we investigate a realization of this model for a robotic system.

## 4. Precision-based attention for Robotics

The previous section introduced a conceptual account to explain the computational mechanisms that undergird attention based on neuroscience findings. We focused on reclaiming saliency as an active process that relies on neural gain control, uncertainty minimization and structured scheduling. Here, we describe how we can mathematically realize some of these mechanisms in the context of well-known challenges in robotics. Enabling robots with this type of attention may be crucial to filter the sensory signals and internal variables that are relevant to estimate the robot/world state and complete any task. More importantly, the active component of salience (i.e., behavior) is essential to interact with the world—as argued in active perception approaches (Bajcsy et al., 2018).

We revisit the standard view of attention in robotics by introducing sensory precision (inverse variance) as the driving mechanism for modulating both perception and action (Friston et al., 2011; Clark, 2013). Although saliency was originally described to underwrite behavior, most models used in robotics, strongly biased by computer vision approaches, focus on computing the most relevant region of an image (Borji and Itti, 2012)—mainly computing human fixation maps—relegating action to a secondary process. Illustratively, state-of-the-art deep learning saliency models—as shown in the MIT saliency benchmark (Bylinskii et al., 2019)—do not have the action as an output. Conversely, the active perception approach properly defines the action as an essential process of active sensing to gather the relevant information. Our proposed model, based on precision modulated action and perception coupling (*i*) place attention as essential for state-estimation and system identification and (*ii*) and reclaims saliency as a driver for information-seeking behavior, as proposed in early works (Tsotsos et al., 1995), but goes beyond human fixation maps for both improving the model of the environment (exploration) and solving the task (exploitation).

In what follows, we highlight the key role of precision by reviewing relevant brain-inspired attention models deployed in robotics (Section 4.1). We propose precision-modulated attentional mechanisms for robots in three contexts—perception (Section 4.2), action (Section 4.3) and active perception (Section 4.3.3). The precision-modulated perception is formalized for a robotics setting; *via* (*i*) state estimation (i.e., estimating the hidden states of a dynamic system from sensory signals—Section 4.2.2), and (*ii*) system identification (i.e., estimating the parameters of the dynamic system from sensory signals—Section 4.2.3). Next, we show that precision-modulated action can be realized through precision optimization (planning future actions—Section 4.3.2) and discuss practical considerations for coupling with precision-modulated perception (precision based active perception—Section 4.3.3). Table 1 summarizes our proposed precision manipulations to solve relevant problems in robot perception and action. Table 2 provides the definitions of precision within our mechanism.

**Table 1:** Robotics applications and their precision realizations.

**Task**	**Application**	**Precision manipulation**	**Sections**
Perception	State and input estimation	Noise precision modeling $\tilde{\Pi }$	4.2.2
	System Identification	Posterior parameter precision learning Π^θ^	4.2.3
	Exploration-exploitation in learning	Prior parameter precision modeling *P*^θ^	4.2.4
	Noise estimation	Noise precision learning $\tilde{\Pi }$	4.2.5
Action	Informative Path Planning (IPP)	Precision optimization (of map)	4.3.2
Active perception	IPP with action-perception cycle	Precision modulation	4.3.3

**Table 2:** Precision parameters that are manipulated in Section 4.2.

**Term**	**Symbol**	**Definition**
Sensory precision	Π^*z*^	Inverse covariance of sensory noise **z** (Equation 1).
Prior parameter precision	*P* ^θ^	The robot's confidence on its prior parameters η^θ^.
Noise precision	$\tilde{\Pi }$	The inverse covariance of all noises (Equation 5).
Posterior parameter precision	Π^θ^	The robot's confidence on its parameter estimates.

### 4.1. Previous brain-inspired attention models in robotics

Brain-inspired attention has been mainly addressed in robotics from a “passive” visual saliency perspective, e.g., which pixels of the image are the most relevant. This saliency map is then generally used to foveate the most salient region. This approach was strongly influenced by early computational models of visual attention (Tsotsos et al., 1995; Itti and Koch, 2001). The first models deployed in robots were bottom-up, where the sensory input was transformed into an array of values that represents the importance (or salience) of each cue. Thus, the robot was able to identify which region of the scene has to look at, independently of the task performed—see Borji and Itti (2012) for a review on visual saliency. These models have also been useful for acquiring meaningful visual features in applications, such as object recognition (Orabona et al., 2005; Frintrop, 2006), localization, mapping and navigation (Frintrop and Jensfelt, 2008; Roberts et al., 2012; Kim and Eustice, 2013). Saliency computation was usually employed as a helper for the selection of the relevant characteristics of the environment to be encoded. Thus, reducing the information needed to process.

More refined methods of visual attention employed top-down modulation, where the context, task or goal bias the relevance of the visual input. These methods were used, for instance, to identify humans using motion patterns (Butko et al., 2008; Morén et al., 2008). A few works also focused on object/target search applications, where top-down and bottom-up saliency attention were used to find objects or people in a search and rescue scenario (Rasouli et al., 2020).

Attention has also been considered in human-robot interaction and social robotics applications (Ferreira and Dias, 2014), mainly for scene or task understanding (Kragic et al., 2005; Ude et al., 2005; Lanillos et al., 2016), and gaze estimation (Shon et al., 2005) and generation (Lanillos et al., 2015a). For instance, computing where the human is looking at and where the robot should look at or which object should be grasped. Furthermore, multi-sensory and 3D saliency computation has also been investigated (Lanillos et al., 2015b). Finally, more complex attention behaviors, particularly designed for social robotics and based on human non-verbal communication, such as joint attention, have also been addressed. Here the robot and the human share the attention of one object through meaningful saccades, i.e., head/eye movements (Nagai et al., 2003; Kaplan and Hafner, 2006; Lanillos et al., 2015a).

Although attention mechanisms have been widely investigated in robotics, specially to model visual cognition (Kragic et al., 2005; Begum and Karray, 2010), the majority of the works have treated attention as an extra feature that can help the visual processing, instead of a crucial component needed for the proper functioning of the cognitive abilities of the robot (Lanillos and Cheng, 2018a). Furthermore, these methods had the tendency to leave the action generation out of the attention process. One of the reasons for not including saliency computation, in robotic systems, is that the majority of the models only output “human-fixation map” predictions, given a static image. Saliency computation introduces extra computational complexity, which can be finessed by visual segmentation algorithms (e.g., line detectors in autonomous navigation). However, it does not resolve uncertainty nor select actions that maximize information gain in the future. In essence, the incomplete view of attention models that output human-fixation maps has arguably obscured the huge potential of neuroscience-inspired attentional mechanisms for robotics.

Our proposed model of attention, based on precision modulation, abandons the current robotics narrow view of attention and saliency by explicitly modeling attention within state estimation, learning and control. Thus, placing attentional processes at the core of the robot computation and not as an extra add-on. In the following sections, we describe the realization of our precision-based attention formulation in robotics using common practical applications as the backbone motif.

### 4.2. Precision-modulated perception

We formalize precision-modulated perception from a first principles Bayesian perspective—explicitly the free energy principle approach proposed by Friston et al. (2011). Practically, this entails optimizing precision parameters over (particular) model parameters.

Through numerical examples show how our model is able to perform accurate state estimation (Bos et al., 2021) and stable parameter learning (Meera and Wisse, 2021a,b). To illustrate the approach, we first introduce a dynamic system modeled as a linear state space system in robotics (Section 4.2.1)—we used this formulation in all our numerical experiments. We briefly review the formal terminologies for a robotics context to appropriately situate our precision-based mechanism for perception. Explicitly, we introduce: precision modeling (by adapting a known form of the precision matrix), precision learning (by learning the full precision matrix), and precision optimization (use precision as an objective function during learning). As a reminder, precision modeling is associated with (instantaneous) gain control and precision learning (at slower time scales) is associated with optimizing that control.

#### 4.2.1. Precision for state space models

A linear dynamic system can be modeled using the following state space equations (boldface notation denotes components of the real system and non-boldface notation its estimates):


(1) $$\begin{array}{r} \begin{array}{c} \overset{∙}{\text{x}}=\text{Ax}+\text{Bu}+\text{w}, \end{array}\;\begin{array}{c} \text{y}=\text{Cx}+\text{z}. \end{array} \end{array}$$

where **A**, **B** and **C** are constant matrices defining the system parameters, **x** ∈ ℝ^*n*^ is the system state (usually an unobserved variable), **u** ∈ ℝ^*r*^ is the input or control actions, **y** ∈ ℝ^*m*^ is the output or the sensory measurements, **w** ∈ ℝ^*n*^ is the process noise with precision **Π^*w*^** (or inverse variance **Σ**^***w***−1^), and **z** ∈ ℝ^*m*^ is the measurement noise with precision **Π^*z*^**.

**Figure 2:**
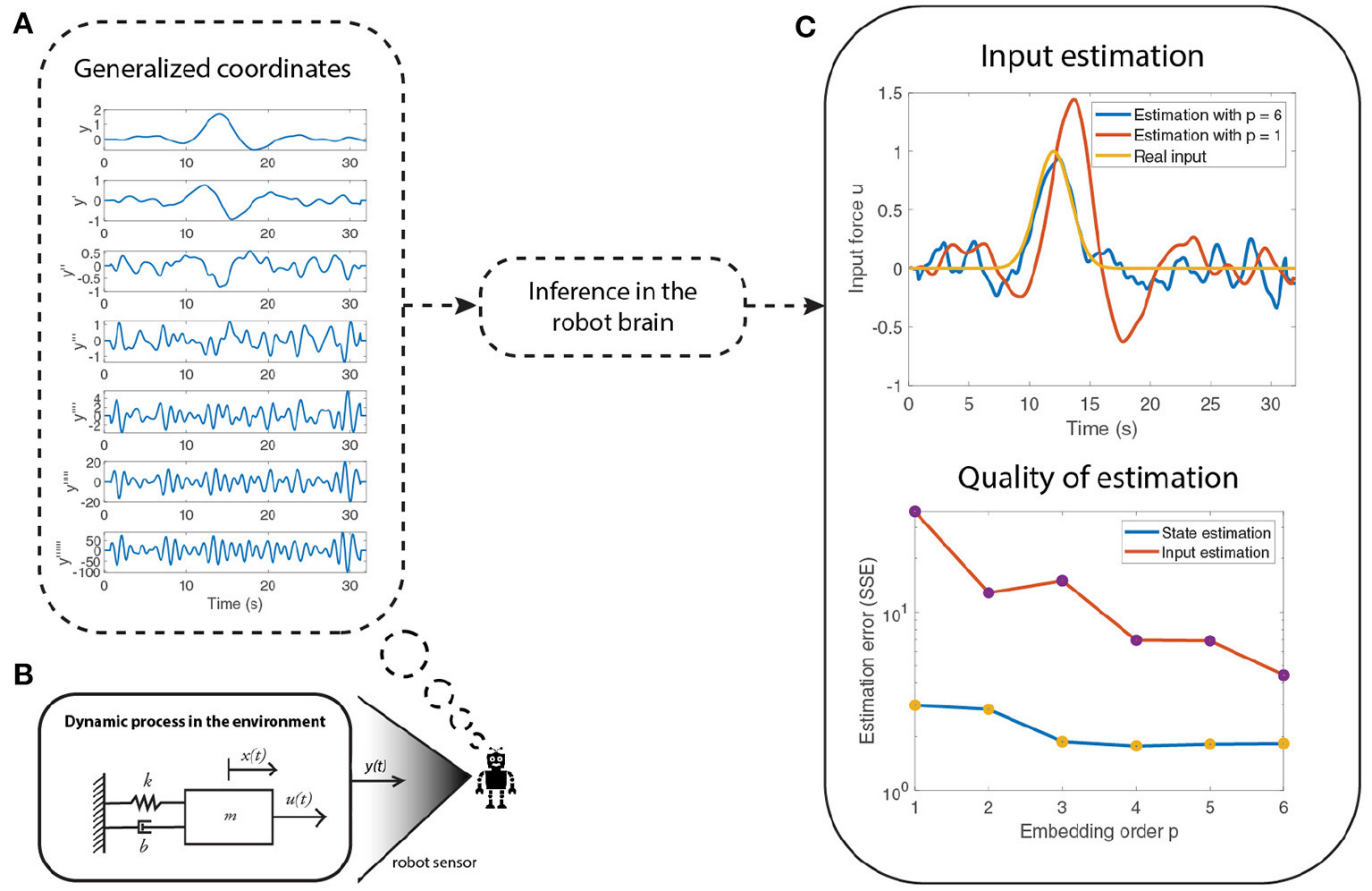
An illustration of an attention mechanism for state and input estimation of a system (shown in **B**). The quality of the estimation improves **(C)** as the embedding order (number of derivatives) of generalized coordinates are increased **(A)**. However, the imprecise information in the higher order derivatives of the sensory input **y** does not affect the final performance of the observer because of attentional selection, which selectively weighs the importance afforded to each derivative, in the free energy optimization scheme.

For instance, we can describe a mass-spring damper system (depicted in Figure 2B) using state space equations. A mass (*m* = 1.4*kg*) is attached to a spring with elasticity constant (*k* = 0.8*N*/*m*), and a damper with a damping coefficient (*b* = 0.4*Ns*/*m*). When a force (*u*(*t*) = *e*^−0.25(*t*−12)^2^^) is applied on the mass, it displaces *x* from its equilibrium point. The linear dynamics of this system is given by:


(2) $$\begin{array}{r} \left[\begin{array}{c} ẋ\\ ẍ \end{array}\right]=\left[\begin{array}{cc} 0 & 1\\ -\frac{k}{m} & -\frac{b}{m} \end{array}\right]\left[\begin{array}{c} x\\ ẋ \end{array}\right]+\left[\begin{array}{c} 0\\ \frac{1}{m} \end{array}\right]u,\;y=\left[\begin{array}{cc} 1 & 0 \end{array}\right]\left[\begin{array}{c} x\\ ẋ \end{array}\right]. \end{array}$$

Note that Equation (2) is equivalent to Equation (1) with parameters **A** $=\left[\begin{array}{cc} 0 & 1\\ -\frac{k}{m} & -\frac{b}{m} \end{array}\right],$**B** =${\left[\begin{array}{c} 0,\frac{1}{m} \end{array}\right]}^{T}$ and **C** $=\left[\begin{array}{cc} 1 & 0 \end{array}\right],$ and state x = ${\left[\begin{array}{c} x,ẋ \end{array}\right]}^{T}$.

Now we introduce attention as precision modulation assuming that the robotic goal is to minimize the prediction error (Friston et al., 2011; Lanillos and Cheng, 2018b; Meera and Wisse, 2020), i.e., to refine its model of the environment and perform accurate state estimation, given the information available. In other words, the robot has to estimate **x** and **u** from input prior η^*u*^ with a prior precision of *P*^*u*^, given the measurements **y**, parameters **A**, **B**, **C** and noise precision **Π^w^** and **Π^z^**. Formally, the prediction error $\tilde{\epsilon }$ of the sensory measurements ${\tilde{\epsilon }}^{y}$, control input reference ${\tilde{\epsilon }}^{u}$ and state ${\tilde{\epsilon }}^{x}$ are:


(3) $$\begin{array}{r} \tilde{\epsilon }=\left[\begin{array}{c} {\tilde{\epsilon }}^{y}\\ {\tilde{\epsilon }}^{u}\\ {\tilde{\epsilon }}^{x} \end{array}\right]=\left[\begin{array}{c} \tilde{\text{y}}-\tilde{\text{C}}\tilde{x}\\ ũ-{\tilde{\eta }}^{u}\\ D^{x}\tilde{x}-\tilde{\text{A}}\tilde{x}-\tilde{\text{B}}ũ \end{array}\right]\;\{\begin{array}{c} \text{sensory prediction error}\\ \text{control input prediction}\\ \text{error}\\ \text{state prediction error} \end{array} \end{array}$$

Note that ${\tilde{\epsilon }}^{y}=\tilde{\text{y}}-\tilde{\text{C}}\tilde{x}$ is the difference between the observed measurement and the predicted sensory input given the state^3^. Here *D*^*x*^ performs the (block) derivative operation, which is equivalent to shifting up all the components in generalized coordinates by one block.

We can estimate the state and input using the Dynamic Expectation Maximization (DEM) algorithm (Friston et al., 2008; Meera and Wisse, 2020) that optimizes a free energy variational bound F to be tractable^4^. This is:


(4) $$\begin{array}{r} X=\left[\begin{array}{c} \tilde{x}\\ ũ \end{array}\right]=\arg\underset{X}{\max}F=\arg\underset{X}{\max}-\frac{1}{2}{\tilde{\epsilon }}^{T}\tilde{\Pi }\tilde{\epsilon } \end{array}$$

Crucially, $\tilde{\Pi }$ is the generalized noise precision that modulates the contribution of each prediction error to the estimation of the state and the computation of the action. Thus, $\tilde{\Pi }$ is equivalent to attentional gain. For instance, we can model the precision matrix to attend to the most informative signal derivatives in $\tilde{\text{y}}$. Concisely, the precision $\tilde{\Pi }$ has the following form:


(5) $$\begin{array}{r} \tilde{\Pi }=\left[\begin{array}{ccc} S⊗\Pi^{z} & 0 & 0\\ 0 & S⊗P^{u} & 0\\ 0 & 0 & S⊗\Pi^{w} \end{array}\right], \end{array}$$

where *S* is the smoothness matrix. In Section 4.2.2, we show that modeling the precision matrix $\tilde{\Pi }$ using the *S* matrix improves the estimation quality.

The full free energy functional (time integral of free energy $\bar{F}=\int Fdt$ at optimal precision) that the robot optimizes to perform state-estimation and system identification is described in Equation (6)—for readability we omitted the details of the derivation of this cost function, and we refer to Anil Meera and Wisse (2021) for further details.


(6) $$\begin{array}{l} ℱ=-\frac{1}{2}\sum_{t}[\underset{\text{precision weighed prediction error}}{\underset{︸}{{\tilde{\epsilon }}^{yT}{\tilde{\Pi }}^{z}{\tilde{\epsilon }}^{y}+{\tilde{\epsilon }}^{uT}P^{\tilde{u}}{\tilde{\epsilon }}^{u}+{\tilde{\epsilon }}^{xT}{\tilde{\Pi }}^{w}{\tilde{\epsilon }}^{x}}}]-\frac{1}{2}[\underset{\text{prior precision weighed prediction error of }\theta \text{ and }\lambda }{\underset{︸}{\epsilon^{\theta T}P^{\theta }\epsilon^{\theta }+\epsilon^{\lambda T}P^{\lambda }\epsilon^{\lambda }}}]\\ \;+\underset{\text{state and input entropy}}{\underset{︸}{\frac{1}{2}n_{t}\ln|\Sigma^{X}|}}+\frac{1}{2}n^{t}\underset{\text{noise entropy}}{\underset{︸}{\left[\ln|{\tilde{\Pi }}^{z}|+\ln|P^{\tilde{v}}|+\ln|{\tilde{\Pi }}^{w}|\right]}}+\underset{\text{parameter entropy}}{\underset{︸}{\frac{1}{2}\ln|\Sigma^{\theta }P^{\theta }|}}+\underset{\text{hyperparameter entropy}}{\underset{︸}{\frac{1}{2}\ln|\Sigma^{\lambda }P^{\lambda }|}} \end{array}$$

Here ϵ^θ^ = θ−η^θ^, ϵ^λ^ = λ−η^λ^ are the prediction errors of parameters and hyper-parameters^5^. $\bar{F}$ consist of two main components: i) precision weighed prediction errors and ii) precision-based entropy. The dominant role of precision—in the free energy objective—is reflected in how modulating these precision parameters can have a profound influence perception and behavior. The theoretical guarantees for stable estimation (Meera and Wisse, 2021b), and its application on real robots (Lanillos et al., 2021) make this formulation very appealing to robotic systems.

Note that we can manipulate three kinds of precision within the state space formulation: (i) prior precision (*P*^ũ^, *P*^θ^, *P*^λ^), (ii) conditional precision on estimates (Π^*X*^, Π^θ^, Π^λ^) and (iii) noise precision (Π^*z*^, Π^*w*^). Therefore, to learn the correct parameter values θ, we (i) learn the parameter precision Π^θ^, (ii) model the prior parameter precision *P*^θ^, and (iii) learn the noise precision Π^*w*^ and Π^*z*^ (parameterised using λ).

#### 4.2.2. State and input estimation

State estimation is the process of estimating the unobserved states of a real system from (noisy) measurements. Here, we show how we can achieve accurate estimation through precision modulation in a linear time invariant system under the influence of colored noise (Meera and Wisse, 2020). State estimation in the presence of colored noise is inherently challenging, owing to the non-white nature of the noise, which is often ignored in conventional approaches, such as the Kalman Filter (Welch and Bishop, 2002).

Figure 2 summarizes a numerical example that shows how one can use precision modulation to focus on the less noisy derivatives (lower derivatives) of measurements, relative to imprecise higher derivatives. Thus, enabling the robot to use the most informative data for state and input estimation, while discarding imprecise input. Figure 2B depicts the mass-spring damper system used. The numerical results show that the quality of the estimation increases as the embedding ordering increases but the lack of information in the higher order derivatives of the sensory input do not affect the final performance due to the precision modulation. The higher order derivatives (Figure 2A) are less precise than the lower derivatives, thereby reflecting the loss of information in higher derivatives. The state and input estimation was performed using the optimization framework described in the previous section. The quality of estimation is shown in Figure 2C, where the input estimation using six derivatives (blue curve) is closer to the real input (yellow curve) than when compared to the estimation using only one derivative (red curve). The quality of the estimation reports the sum of squared error (SSE) in the estimation of states and inputs with respect to the embedding order (number of signal derivatives considered).

To obtain accurate state estimation by optimizing the precision parameters, we recall that the precision weights the prediction errors. From Equation (3), the structural form of $\tilde{\Pi }$ is mainly dictated by the smoothness matrix *S*, which establishes the interdependence between the components of the variable expressed in generalized coordinates (e.g., the dependence between **y**, **y**′ and **y**″ in $\tilde{\text{y}}$). For instance, the *S* matrix for a Gaussian kernel is as follows Meera and Wisse (2022):


(7) $$\begin{array}{r} S=\left[\begin{array}{ccccccc} \frac{35}{16} & 0 & \frac{35}{8}s^{2} & 0 & \frac{7}{4}s^{4} & 0 & \frac{1}{6}s^{6}\\ 0 & \frac{35}{4}s^{2} & 0 & 7s^{4} & 0 & s^{6} & 0\\ \frac{35}{8}s^{2} & 0 & \frac{77}{4}s^{4} & 0 & \frac{19}{2}s^{6} & 0 & s^{8}\\ 0 & 7s^{4} & 0 & 8s^{6} & 0 & \frac{4}{3}s^{8} & 0\\ \frac{7}{4}s^{4} & 0 & \frac{19}{2}s^{6} & 0 & \frac{17}{3}s^{8} & 0 & \frac{2}{3}s^{10}\\ 0 & s^{6} & 0 & \frac{4}{3}s^{8} & 0 & \frac{4}{15}s^{10} & 0\\ \frac{1}{6}s^{6} & 0 & s^{8} & 0 & \frac{2}{3}s^{10} & 0 & \frac{4}{45}s^{12} \end{array}\right], \end{array}$$

where *s* is the kernel width of the Gaussian filter that is assumed to be responsible for serial correlations in measurement or state noise. Here, the order of generalized coordinates (number of derivatives under consideration) is taken as six (*S* ∈ ℝ^7 × 7^). For practical robotics applications, the measurement frequency is high, resulting in 0 < *s* < 1. It can be observed that the diagonal elements of *S* decreases because *s* < 1, resulting in a higher attention (or weighting) on the prediction errors from the lower derivatives when compared to the higher derivatives. The higher the noise color (i.e., *s* increases), the higher the weight given to the higher state derivatives (last diagonal elements of *S* increases). This reflects the fact that smooth fluctuations have more information content in their higher derivatives. Having established the potential importance of precision weighting in state estimation, we now turn to the estimation (i.e., learning) of precision in any given context.

#### 4.2.3. System identification

This section shows how to optimize system identification by means of precision learning (Anil Meera and Wisse, 2021; Meera and Wisse, 2021b). Specifically, we show how to fuse prior knowledge about the dynamic model with the data to recover unknown parameters of the system through an attention mechanism. This involves the learning of the (1) parameters and (2) noise precisions. Our model “turns” the attention to the least precise parameters and uses the data to update those parameters to increase their precision. Hence, allowing faster parameter learning.

For the sake of clarity, we use again the mass-spring-damper system as the driving example (Section 4.2.1). We formalize system identification as evaluating the unknown parameters *k*, *m* and *b*, given the input **u**, the output **y**, and the general form of the linear system in Equation (2).

**Figure 3:**
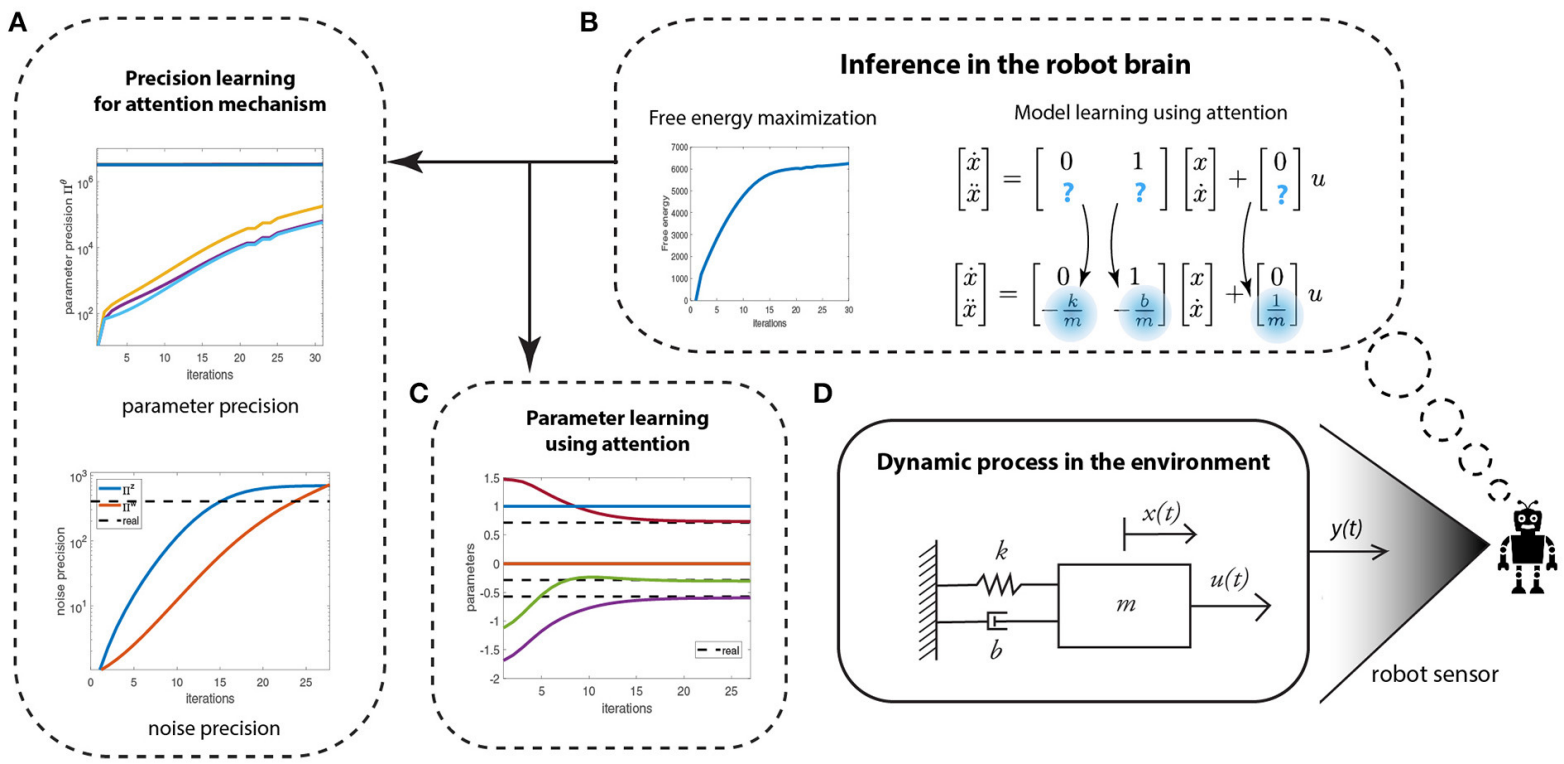
The schematic of the robot's attention mechanism for learning the least precise parameters of a given generative model of a mass-spring-damper system (shown in **D**). **(A)** Learning the conditional precision on parameters and the noise precision. **(B)** The free energy optimization helping to identify the unknown system parameters. **(C)** The parameter learning.

Figure 3 depicts the process of learning unknown parameters (dotted boxes denote the processes inside the robot brain). The robot measures its position *x*(*t*) using its sensors (e.g., vision or range sensor). We assume that the robot has observed the behavior of a mass-spring-damper system before or a model is provided by the expert designer. However, some of the parameters are unknown. The robot can reuse the prior learned model of the system to relearn the new system. This can be realized by setting a high prior precision on the known parameters and a low prior precision on the unknown parameters. By means of precision learning, the robot uses the sensory signals to learn the parameter precision Π^θ^, thereby improving the confidence in the parameter estimates θ. This directs the robot's attention toward the refinement of the parameters with least precision as they are the most uncertain. The requisite parameter learning proceeds by the gradient ascent of the free energy functional given in Equation (6). The parameter precision learning proceeds by tracking the negative curvature of $\bar{F}$ as $\Pi^{\theta }=-\frac{\partial^{2}\bar{F}}{\partial \theta^{2}}$ (Anil Meera and Wisse, 2021).

The learning process—by means of variational free energy optimization (maximization)—is shown in Figure 3B. The learning involves two parallel processes: precision learning (Figure 3A), and parameter learning (Figure 3C). Precision learning comprises of parameter precision learning (top graph)—i.e., identifying the precision of an approximate posterior density for the parameters being estimated—and noise precision learning (bottom graph). The high prior precision on the known system parameters (0 and 1), and low prior precision on the unknown system parameters ($-\frac{k}{m},-\frac{b}{m}$ and $\frac{1}{m}$, highlighted in blue) directs attention toward learning the unknown parameters and their precision. Note that in Figure 3A, the precision on the three unknown parameters start from a low prior precision of *P*^θ^ = 1 and increase with each iteration, whereas the precision of known parameters (0 and 1) remains a constant (3.3 × 10^6^). The noise precisions are learned simultaneously, which starts from a low prior precision of *P*^λ^^*w*^ = *P*^λ^^*z*^ = 1 and finally converges to the true noise precision (dotted black line). Both precisions are used to learn the three parameters of the system (Figure 3B), which starts from randomly selected values within the range [−2,2] and finally converges to the true parameter values of the system ($\theta_{3}=-\frac{k}{m}=-0.5714$, $\theta_{4}=-\frac{b}{m}=-0.2857$ and $\theta_{6}=\frac{1}{m}=0.7143$), denoted by black dotted lines. From an attentional perspective, the lower plot in Figure 3A is particularly significant here. This is because the robot discovers the data are more informative than initially assumed, thereby leading to an increase in its estimate of the precision of the data-generating process. This means that the robot is not only using the data to optimize its beliefs about states and parameters (system identification), it is also using these data to optimize the way in which it assimilates these data.

In summary, precision-based attention, in the form of precision learning, helps the robot to accurately learn unknown parameters by fusing prior knowledge with new incoming data (sensory measurements), and attending to the least precise parameters.

#### 4.2.4. Precision-modulated exploration and exploitation in system identification

Exploration and exploitation in the parameter space can be advantageous to robots during system identification. Precision-based attention—here the prior precision—allows a graceful balance between the two, mediated by the prior precision^6^. A very high prior precision encourages exploitation and biases the robot toward believing its priors, while a low prior precision encourages exploration and makes the robot sensitive to new information.

**Figure 4:**
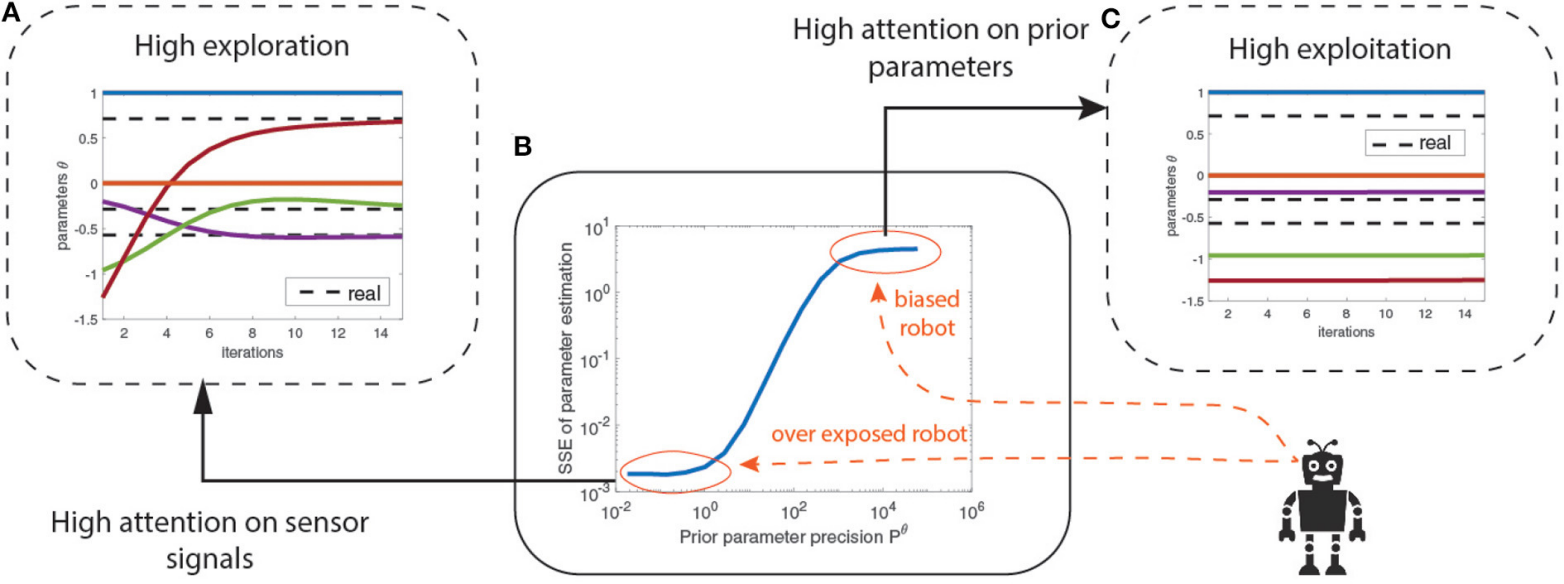
**(A)** Lower *P*^∧^θ gives a high exploration strategy across the parameter space. **(B)** Precision-based attention allows exploration and exploitation balanced model learning mediated by the prior precisions on the parameters *P*^∧^θ. **(C)** The higher the *P*^∧^θ, the higher the attention on prior parameters η^∧^θ and the lower the attention on the sensory signals while learning.

We use again the mass-spring-damper system example but with a different prior parameter precision *P*^θ^. The prior parameters are initialized at random and learned using optimization. Figure 4B shows the increase in parameter estimation error (SSE) as the prior parameter precision *P*^θ^ increases until it finally saturates. The bottom left region (circled in red) indicates the region where the prior precision is low, encouraging exploration with high attention on the sensory signals for learning the model. This region over-exposes the robot to its sensory signals by neglecting the prior parameters. The top right region (circled in red) indicates the biased robot where the prior precision is high, encouraging the robot to exploit its prior beliefs by retaining high attention on prior parameters. This regime biases the robot into being confident about its priors and disregarding new information from the sensory signals. Between those extreme regimes (blue curve) the prior precision balances the exploration-exploitation trade-off. Figure 4A describes how increased attention to sensory signals helped the robot to recover from poor initial estimates of parameter values and converge toward the correct values (dotted black line). Conversely, in Figure 4C, high attention on prior parameters did not help the robot to learn the correct parameter values.

These results establish that prior precision modeling allows balanced exploration and exploitation of parameter space during system identification. Although the results show that an over-exposed robot provides better parameter learning, we show—in the next section—that this is not always be the case.

#### 4.2.5. Noise estimation

In real-world applications, sensory measurements are often highly noisy and unpredictable. Furthermore, the robot does not have access to the noise levels. Thus, it needs to learn the noise precision (**Π^*z*^**) for accurate estimation and robust control. Precision-based attention enables this learning. In what follows, we show how one can estimate **Π^*z*^** using noise precision learning and that biasing the robot to prior beliefs can be advantageous in highly noisy environments.

**Figure 5:**
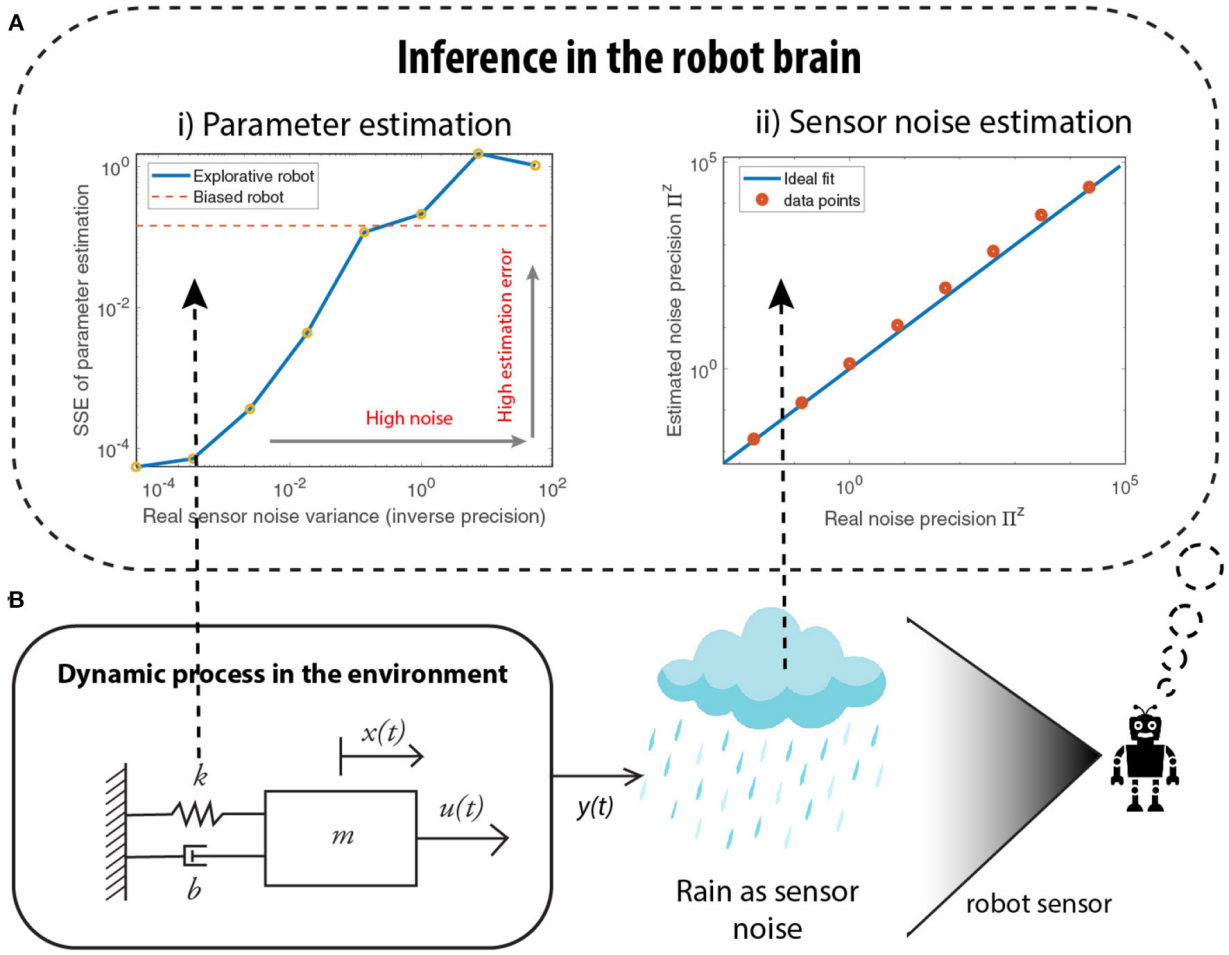
Simulations demonstrating how a biased robot could be advantageous, especially while learning in a highly noisy environment (shown in **B**). **(A i)** As the sensor noise increases, the quality of parameter estimation deteriorates to a point where an explorative robot generates higher parameter estimation errors than when compared to the biased robot that relies on its prior parameters. **(A ii)** However, the sensor noise estimation is accurate even for high noise environments, demonstrating the success of the attention mechanism using the noise precision.

Consider again the mass-spring-damper system in Figure 5B, where heavy rainfall/snow corrupts visual sensory signals. We evaluate the parameter estimation error under different noise conditions, using different levels of noise variances (inverse precision). For an over-exposed robot (only attending to sensory measurements), left plot of Figure 5A, the estimation error increases as the noise strength increases, to a point where the error surpasses the error from a prior-biased robot. This shows that a robot, confident in its prior model, assigns low attention to sensory signals and outperforms an over-exposed robot that assigns high attention to sensory signals, in a highly noisy environment. The right plot of Figure 5A shows the quality of noise precision learning for an over-exposed robot. It can be seen that all the data points in red lie close to the blue line, indicating that the estimated noise precision is close to the real noise precision. Therefore, the robot is capable of recovering the correct sensory noise levels even when the environment is extremely noisy, where accurate parameter estimation is difficult.

These numerical results show that attention mechanism—by means of noise precision learning—allows the estimation of the noise levels in the environment and thereby protects against over-fitting or overconfident parameter estimation.

**Summary**. We have shown how precision-based attention–through precision modeling and learning– yields to accurate robot state estimation, parameter identification and sensory noise estimation. In the next section, we discuss how action is generated in this framework.

### 4.3. Precision-modulated action

Selecting the optimal sequence of actions to fulfill a task is essential for robotics (LaValle, 2006). One of the most prominent challenges is to ensure robust behavior given the uncertainty emerging from a highly complex and dynamic real world, where the robots have to operate on. A proper attention system should provide action plans that resolve uncertainty and maximize information gain. For instance, it may minimize the information entropy, thereby encouraging repeated sensory measurements (observations) on high uncertainty sensory information.

Salience, which in neuroscience is sometimes identified as Bayesian surprise (i.e., divergence between prior and posterior), describes which information is relevant to process. We go one step further by defining the saliency map as the epistemic value of a particular action (Friston et al., 2015). Thus, the (expected) divergence now becomes the mutual information under a particular action or plan. This makes the saliency map more sophisticated because it is an explicit measure of the reduction in uncertainty or mutual information associated with a particular action (i.e., active sampling), and more pragmatic because it tells you where to sample data next, given current Bayesian beliefs.

**Figure 6:**
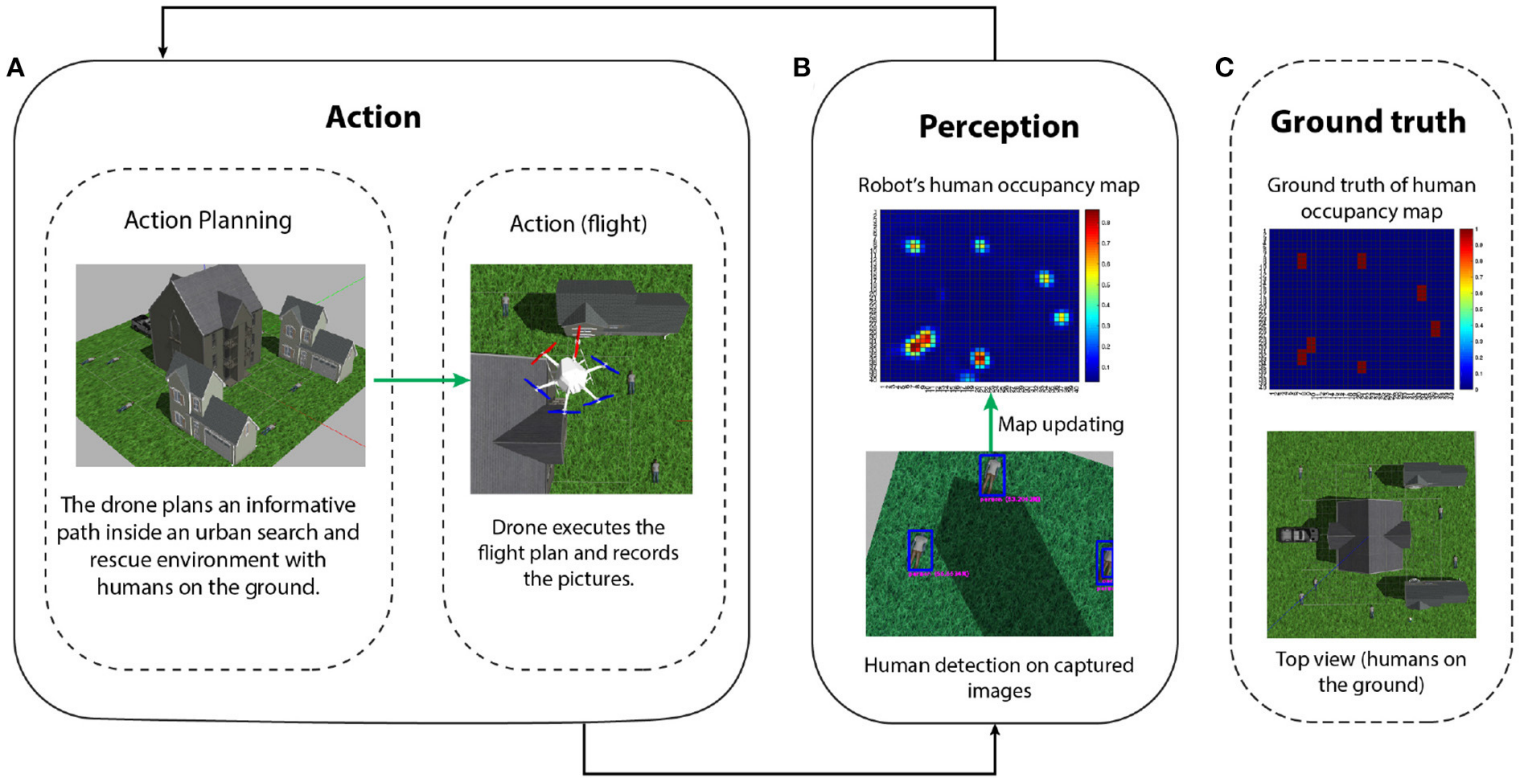
IPP problem for localizing human victims in an urban search and rescue scenario (Meera et al., 2019). **(A)** Action: a UAV, in a realistic simulation environment, plans a finite look-ahead path to minimize the uncertainty of its human occupancy map (e.g., modeled as a Gaussian process) of the world. The planned path is then executed, during which the UAV flies and captures images at a constant measurement frequency. **(B)** Perception: after the data acquisition is complete, a human detection algorithm is executed to detect all the humans on the images. These detections are then fused into the UAV's human location map. The cycle is repeated until the uncertainty of the map is completely resolved (this usually implies enough area coverage and repeated measurements on uncertain locations). The ground truth of the human occupancy map and the UAV belief is shown in **(B,C)** respectively. The final map approaches the ground truth and all the seven humans on the ground are correctly detected.

We first describe a precision representation usually used in information gathering problems and then how to directly generate action plans through precision optimization. Afterwards, we discuss the realization of the full-fledged model presented in the neuroscience section for active perception. We use the informative path planning (IPP) problem, described in Figure 6, as an illustrative example to drive intuitions.

#### 4.3.1. Precision maps as saliency

One of the popular approaches in information gathering problems is to model the information map as a distribution [e.g., using Gaussian processes (Hitz et al., 2017)]. This is widely used in applications, such as a target search, coverage and navigation. The robot keeps track of an occupancy map and the associated uncertainty map (covariance matrix or inverse precision). While the occupancy map records the presence of the target on the map, the uncertainty map records the quality of those observations. The goal of the robot is to learn the distribution using some learning algorithm (Marchant and Ramos, 2014). A popular strategy is to plan the robot path such that it minimizes the uncertainty of the map in future (Popović et al., 2017). In Section 4.3.2, we will show how we can use the map precision to perform active perception, i.e., optimize the robot path for maximal information gain. Optimizing the map precision drives the robot toward an exploratory behavior.

#### 4.3.2. Precision optimization for action planning

**Figure 7:**
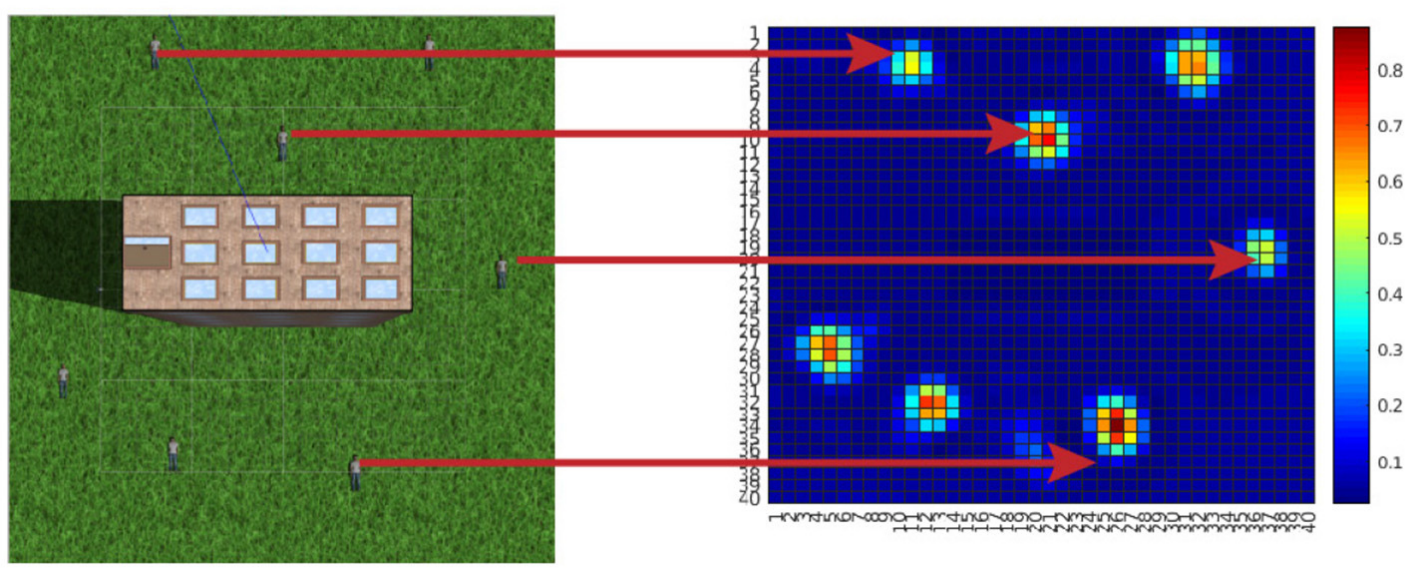
Finding humans with unmanned air vehicles (UAVs): an informative path planning (IPP) approach (Anil Meera, 2018). The simulation environment on the left consists of a tall building at the center, surrounded by seven humans lying on the floor. The goal of the UAV is to compute the action sequence that allows maximum information gathering, i.e., the humans location uncertainty is minimized. On the right is the final occupancy map colored with the probability of finding a human at that location. It can be observed that all humans on the simulation environment were correctly detected by the robot.

**Figure 8:**
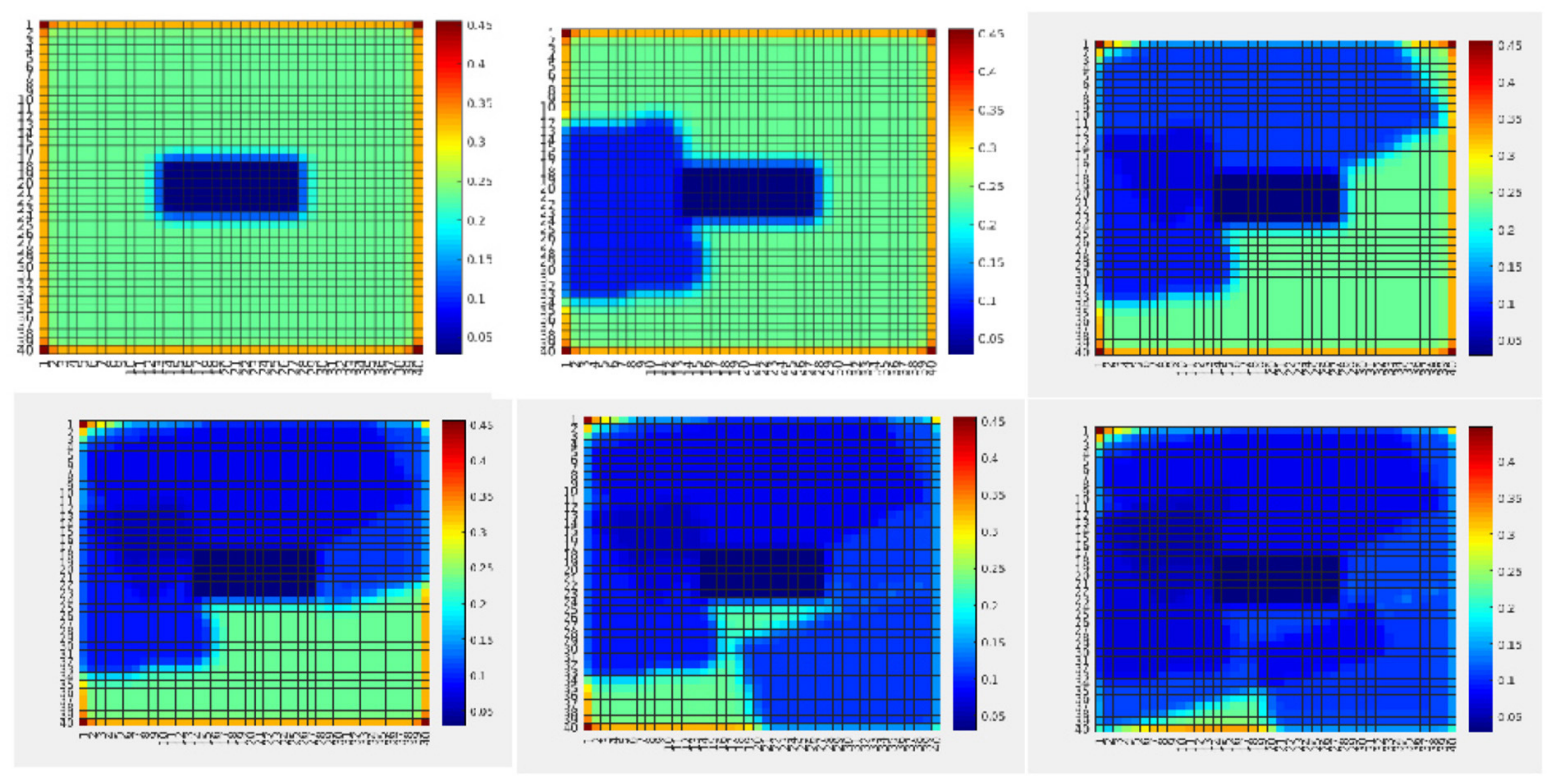
Variance map of the probability distribution of people location (Figure 7)—inverse precision of human occupancy map. The plot sequence shows the reduction of map uncertainty (inverse precision) after measurements (Anil Meera, 2018).

To introduce precision-based saliency we use an exemplary application of search and rescue. The goal is to find all humans using an unmanned air vehicle (UAV) (Lanillos, 2013; Lanillos et al., 2014; Meera et al., 2019; Rasouli et al., 2020). We use precision for two purposes: (i) precision optimization for action planning (plan flight path) and (ii) precision learning for map refinement. In contrast to previous models of action selection within active inference in robotics (Lanillos et al., 2021; Oliver et al., 2021) here precision explicitly drives the agent behavior. Figure 7 describes the scenario in simulation. The seven human targets on the ground are correctly identified by the UAV. We can formalize the solution as the UAV actions (next flight path) that minimize the future uncertainties of the human occupancy map. In our precision-based attention scheme, this objective is equivalent to maximizing the posterior precision of the map. Figure 8 shows the reduction in map uncertainty after subsequent assimilation of the measurements (camera images from the UAV, processed by a human detector). The map (and precision) is learned using a recursive Kalman Filter by fusing the human detector outcome onto the map (and precision). The algorithm drives the UAV toward the least explored regions in the environment, defined by the precision map.

**Figure 9:**
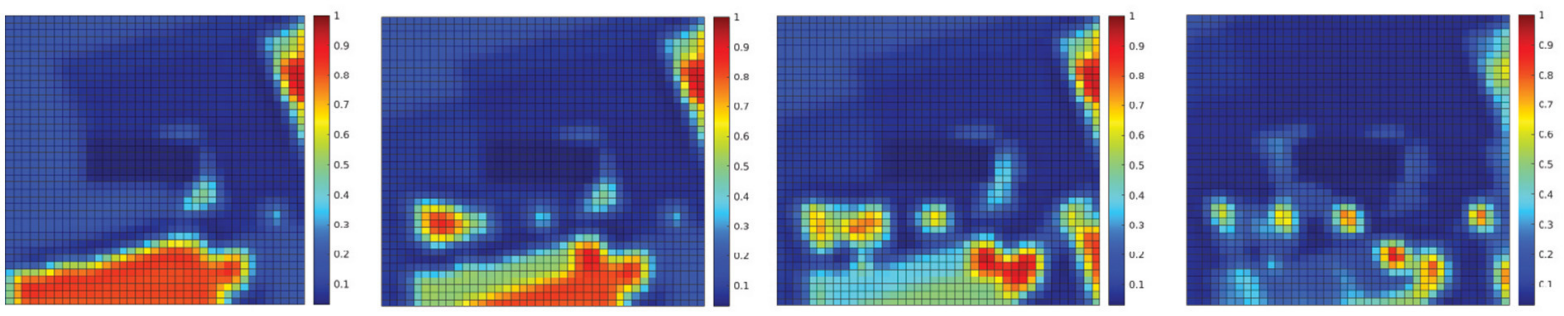
The human occupancy map (probability to find humans at every location of the environment) at four time instances during the UAV flight showing ambiguity resolution. The ambiguity arising from imprecise sensor measurements (false positive) is resolved through repeated measurements at the same location. The plot sequence shows how the assimilation of the measurements updates the probability of the people being in each location of the map (Meera et al., 2019).

Furthermore, Figure 9 shows an example of uncertainty resolution under false positives. In this case, human targets are moved to the bottom half of the map. The first measurement provides a wrong human detection with high uncertainty. However, after repeated measurements at the same location in the map the algorithm was capable of resolving this ambiguity, to finally learn the correct ground truth map. Hence, the sought behavior is to take actions that encourage repeated measurements at uncertain locations for reducing uncertainty.

Although the IPP example illustrates how to generate control actions through precision optimization, the task, by construction, is constrained to explicitly reduce uncertainty. This is similar to the description of visual search described in Friston et al. (2012), where the location was chosen maximize information gain. Information gain (i.e., the Bayesian surprise expected following an action) is a key part of the expected free energy functional that underwrite action selection in active inference. In brief, expected free energy can be decomposed into two parts the first corresponds to the information gain above (a.k.a., epistemic value or affordance). The second corresponds to the expected log evidence or marginal likelihood of sensory samples (a.k.a., pragmatic value). When this likelihood is read as a prior preference, it contextualizes the imperative to reduce uncertainty by including a goal-directed, imperative. For example, in the search paradigm above, we could have formulated the problem in terms of reducing uncertainty about whether each location was occupied by a human or not. We could have then equipped the agent with prior preferences for observing humans.

In principle, this would have produced searching behavior until uncertainty had been resolved about the scene; after which, the robot would seek out humans; simply because, these are its preferred outcomes. In thinking about how this kind of neuroscience inspired or biomimetic approach could be implemented in robotics, one has to consider carefully, the precision afforded sensory inputs (i.e., the likelihood of sensory data, given its latent causes)—and how this changes during robotic flight and periods of data gathering. This brings us back to the precision modulation and the temporal scheduling of searching and securing data. In the final section, we conclude with a brief discussion of how this might be implemented in future applications.

#### 4.3.3. Precision-based active perception

**Figure 10:**
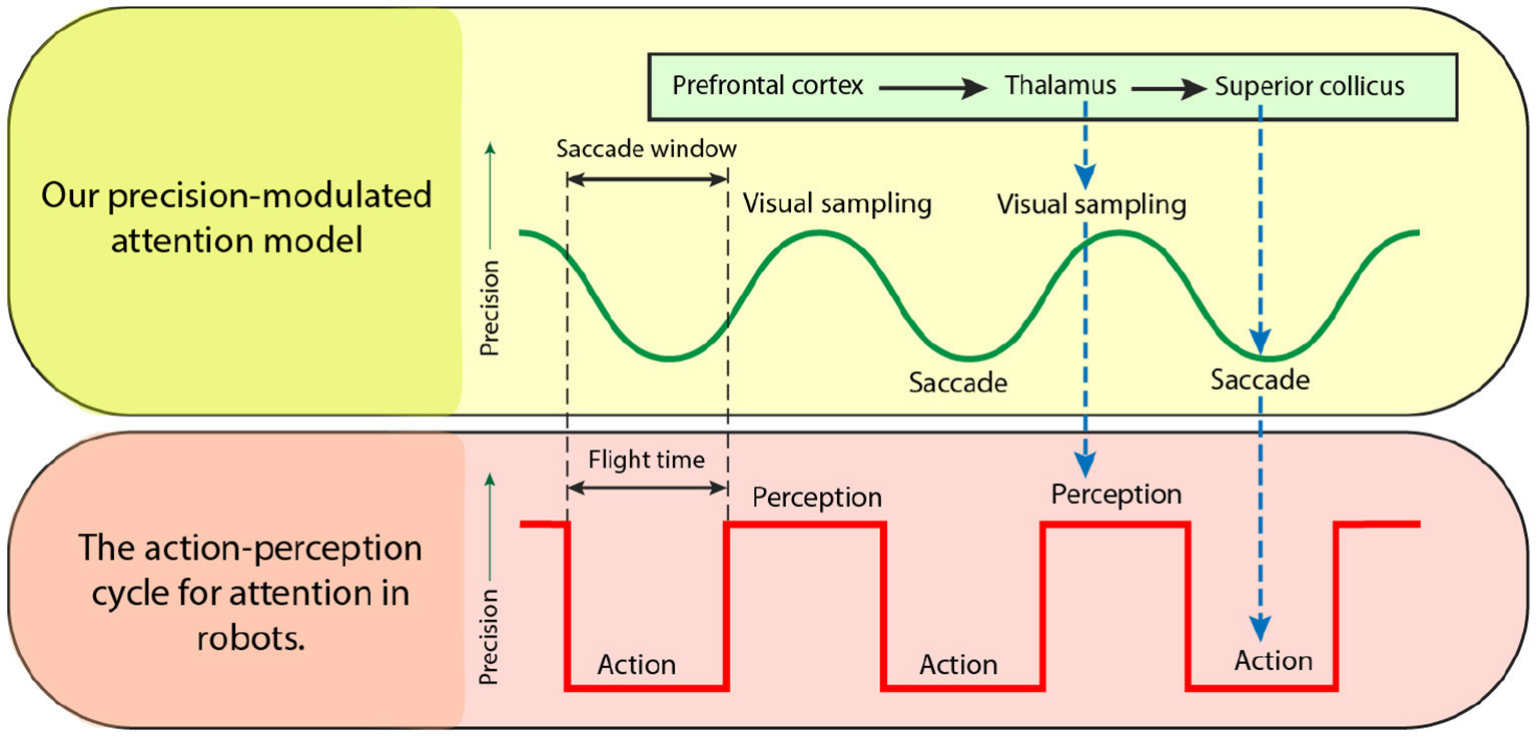
Precision-modulated attention model adapted to the action-perception loop in robotics. Each cycle consists of two steps: (1) action (planning and execution of a finite-time look ahead of the robot path for data collection) and (2) perception (learning using the collected data). This scheduling, using a finite time look-ahead plan, is quite common in real applications and of particular importance when processing is computationally expensive, e.g., slow rate of classification, non-scalable data fusion algorithms, Exponential planners, etc. However, the benefits of incorporating “optimal” scheduled loop driven by precision should be further studied.

In this section, we discuss the realization of a biomimetic brain-inspired model in relation to existing solutions in robotics in the context of path-planning. Figure 10 compares our proposed precision-modulated attention model—from Figure 1—with the action-perception loop widely used in robotics. By analogy with eye saccades to the next visual sample, the UAV flies (action) over the environment to assimilate sensory data for an informed scene construction (perception). Once the flight time of the UAV is exhausted (similar to saccade window of the eye), the action is complete, after which the map is updated, and the next flight path is planned.

In standard applications of active inference, the information gain is supplemented with expected log preferences to provide a complete expected free energy functional (Sajid et al., 2021a). This accommodates the two kinds of uncertainty that actions and choices typically reduce. The first kind of uncertainty is inherent in unknowns in the environment. This is the information gain we have focused on above. The second kind of uncertainty corresponds to expected surprise, where surprise rests upon a priori expected or preferred outcomes. As noted above, equipping robots with both epistemic and pragmatic aspects to their action selection or planning could produce realistic and useful behavior that automatically resolves the exploration-exploitation dilemma. This follows because the expected free energy contains the optical mixture of epistemic (information-seeking) and pragmatic (i.e., preference seeking) components. Usually, after a period of exploration, the preference seeking components predominate because uncertainty has been resolved. Although expected free energy provides a fairly universal objective function for sentient behavior, it does not specify how to deploy behavior and sensory processing optimally. This brings us to the precision modulation model, inspired by neuroscientific considerations of attention and salience.

Hence, there are key differences between biological and robotic implementations of the search behavior. First, the use of oscillatory precision to modulate visual sampling and movement cycles, as opposed to arbitrary discrete action and perception steps currently used in robotics. Second, precision modulation influences both state estimation and action following the same uncertainty reduction principle. Importantly, our salience formulation speaks to selecting future data that reduces this uncertainty. For instance, we have shown—in the information gathering IPP example described in the previous subsection—that by optimizing precision we also optimize behavior.

Hence, there are key differences between biological and robotic implementations of the search behavior. First, the use of oscillatory precision to modulate visual sampling and movement cycles, as opposed to arbitrary discrete action and perception steps currently used in robotics. Second, precision modulation influences both state estimation and action following the same uncertainty reduction principle. Importantly, our salience formulation speaks to selecting future data that reduces this uncertainty. For instance, we have shown—in the information gathering IPP example described in the previous subsection—that by optimizing precision we also optimize behavior.

We argue the potential need and the advantages of realizing precision based temporal scheduling, as described the our brain-inspired model, for two practically relevant test cases: (*i*) learning dynamic models and (*ii*) information seeking applications.

In Section 4.2.4, we have shown how the exploration-exploitation trade-off can be mediated by the prior parameter precision during learning. However, the accuracy-precision curve (Figure 4B) is often practically unavailable due to unknown true parameters values, challenging the modeling of prior precision. An alternative would be to use a precision based temporal scheduling mechanism to alternate between exploration and exploitation by means of a varying *P*^θ^ (similar to Figure 10) during learning, such that system identification is neither biased nor over exposed to sensory measurements. In Figure 5A, we showed how noise levels influence estimation accuracy, and how biasing the robot by modeling *P*^θ^ can be beneficial for highly noisy environments. A precision based temporal scheduling mechanism by means of a varying *P*^θ^ could provide a balanced solution between a biased robot (that exploits its model) and an exploratory one.

Furthermore, temporal scheduling, in the same way that eye saccades are generated, can be adapted for information gathering applications, such as target search, simultaneous localization and mapping, environment monitoring, etc. For instance, introducing precision-modulation scheduling for solving the IPP, and scheduling perception (map learning) and action (UAV flight). Precision modulation will switch between action and perception: when the precision is high, perception occurs (c.f., visual sampling), and when the precision is low, action occurs (c.f., eye movements). This switch, which is often implemented in the robotics literature using a budget for flight time, will be now dictated by precision dynamics.

In short, we have sketched the basis for a future realization of precision-based active perception, where the robot computes the actions to minimize the expected uncertainty. While most attentional mechanisms in robotics are limited to providing a “saliency” map highlighting the most relevant features, our attention mechanism proposes a general scheduling mechanism with action in the loop with perception, both driven by precision.

## 5. Concluding remarks

We have considered attention and salience as two distinct processes that rest upon oscillatory precision control processes. Accordingly, they require particular temporal considerations: attention to reliably estimate latent states from current sensory data and salience for uncertainty reduction regarding future data samples. This formulation addresses visual search from a first principles (Bayesian) account of how these mechanisms might manifest—and the circular causality that undergirds them *via* a rhythmic theta-coupling. Crucially, we have revisited the definition of salience from the visual neurosciences; where it is read as Bayesian surprise (i.e., the Kullback Leibler divergence between prior and posterior beliefs). We took this one step further and defined salience as the expected Bayesian surprise (i.e., epistemic value) of a particular action (e.g., sampling this set of data) (Friston et al., 2017b; Sajid et al., 2021a). Formulating salience as the expected divergence renders it the mutual information under a particular action (or action trajectory) (Friston et al., 2021),—and highlights its role in encoding working memory (Parr and Friston, 2017b). For brevity, our narrative was centered around visual attention and its realization *via* eye movements. However, this model does not strictly need to be limited to visual information processing, because it addresses sensorimotor and auditory processing in general. This means it explains how action and perception can be coupled in other sensory modalities. For instance, Tomassini et al. (2017) showed that visual information is coupled with finger movements at a theta rhythm.

The point of contact with the robotics use of salience emerges because the co-variation between a particular parameterisation and the inputs is a measure of the mutual information between the data and its estimated causes. In this sense, both definitions of salience reflect the mutual information—or information about a particular representation of a (latent) cause—afforded by an observation or consequence. However, our formulation is more sophisticated. Briefly, because it is an explicit measure of the reduction in uncertainty (i.e., mutual information) associated with a particular action (i.e., active sampling) and specifies where to sample data next, given current Bayesian beliefs. These processes (attention and salience) are a consequence of precision of beliefs over distinct model parameters. Explicitly, attention contends with precision over the causes of (current) outcomes and salience contends with beliefs about the data that has to be acquired and precision over beliefs about actions that dictate it. Since both processes can be linked *via* precision manipulation, the crucial thing is the precision that differentiates whether the agent acquires new information (under high precision) or resolves uncertainty by moving (low precision).

The focus of this work has been to illustrate the importance of optimizing precision at various places in generative models used for data assimilation, system identification and active sensing. A key point—implicit in these demonstrations - rests upon the mean field approximation used in all applications. Crucially, this means that getting the precision right matters, because updating posterior estimates of states, parameters and precisions all depend upon each other. This may be particularly prescient for making the most sense of samples that maximizes information gain. In other words, although attention and salience are separable optimization processes, they depend upon each other during active sensing. This was the focus of our final numerical studies of action planning.

To face-validate our formulation, we evaluated precision-modulated attentional processes in the robotic domain. We presented numerical examples to show how precision manipulation underwrites accurate state and noise estimation (e.g., selecting relevant information), as well as allowing system identification (e.g., learning unknown parameters of the dynamics). We also showed how one can use precision-based optimization to solve interesting problems; like the informative path planning in search and rescue scenarios. Thus, in contrast to previous uses of attention in robotics, we placed attention and saliency as integral processes for efficient gathering and processing of sensory information. Accordingly, ‘attention' is not only about filtering the current flow of information from the sensors but performing those actions that minimize expected uncertainty. Still, the full potential of our proposal has yet to be realized, as the precision-based attention should be able to account for prior preferences beyond the IPP problem (e.g., localizing people using UAVs). Finally, we briefly considered the realization of temporal scheduling for information gathering tasks, opening up interesting lines of research to provide robots with biologically plausible attention.

## Data availability statement

The original contributions presented in the study are included in the article/supplementary material, further inquiries can be directed to the corresponding author/s.

## Author contributions

AA and FN are responsible for the novel account and its translation to robotics. AA, FN, PL, and NS wrote the manuscript. All authors contributed to conception and design of the work, manuscript revision, read, and approved the submitted version.

## Funding

The open access publication of the manuscript was funded by the TU Delft Library. FN was funded by the Serotonin & Beyond project (953327). PL was partially supported by Spikeference project, Human Brain Project Specific Grant Agreement 3 (ID: 945539). NS was funded by the Medical Research Council (MR/S502522/1) and 2021-2022 Microsoft Ph.D. Fellowship. KF is supported by funding for the Wellcome Centre for Human Neuroimaging (Ref: 205103/Z/16/Z) and a Canada UK Artificial Intelligence Initiative (Ref: ES/T01279X/1).

## Conflict of interest

The authors declare that the research was conducted in the absence of any commercial or financial relationships that could be construed as a potential conflict of interest.

## Publisher's note

All claims expressed in this article are solely those of the authors and do not necessarily represent those of their affiliated organizations, or those of the publisher, the editors and the reviewers. Any product that may be evaluated in this article, or claim that may be made by its manufacturer, is not guaranteed or endorsed by the publisher.

## Footnotes

^1^Covert attention is where saccadic eye movements do not occur.

^2^Overt attention deals with how an agent tracks the object with eye movements.

^3^The tilde over the variable refers to the generalized coordinates, i.e., the variable includes all temporal derivatives. Thus, $\tilde{\epsilon }$ is the combined prediction error of outputs, inputs and states. For example, the generalized output ỹ is given by $\tilde{\text{y}}={\left[y,y^{\prime },y^{\prime \prime }...\right]}^{T}$, where the prime operator denotes the derivatives. We use generalized coordinates (Friston et al., 2010) for achieving accurate state and input estimation during the presence of (colored) noise by modeling the time dependent quantities (*x, v, y, w, z*) in generalized coordinates. This involves keeping track of the evolution of the trajectory of the probability distributions of states, instead of just their point estimates. Here the colored noise *w* and *z* are modeled as a white noise convoluted with a Gaussian kernel. The use of generalized coordinates has recently shown to outperform classical approaches under colored noise on real quadrotor flight (Bos et al., 2021).

^4^Note that this expression of the variational free energy is using the Laplace and mean-field approximations commonly used in the FEP literature.

^5^System identification involves the estimation of system parameters (denoted by θ, e.g., vectorised **A**), given **y**, **u**, by starting from a parameter prior of η^θ^ with prior precision *P*^θ^, and a prior on noise hyper-parameter η^λ^ with a prior precision of *P*^λ^. Note that we parametrise noise precision (Π^*w*^ and Π^*z*^) using $\lambda \in {\mathbb{R}}^{2\times 1}=\left[\begin{array}{c} \lambda^{z}\\ \lambda^{w} \end{array}\right]$ as an exponential relation (e.g., Π^*w*^(λ^*w*^) = exp(λ^*w*^)*I*^*n*×*n*^).

^6^Note that here we are using exploration and exploration not in terms of behavior but for parameter learning. Exploration means adapting the parameter to a different (unexplored) value and exploitation means keeping that value.

## References

[B1] Ahnelt P. (1998). The photoreceptor mosaic. Eye 12, 531–540. 10.1038/eye.1998.142 9775214

[B2] Anil Meera A. (2018). Informative path planning for search and rescue using a uav (Maters thesis). TU Delft.

[B3] Anil Meera A. Wisse M. (2021). Dynamic expectation maximization algorithm for estimation of linear systems with colored noise. Entropy 23, 1306. 10.3390/e23101306 34682030 PMC8534782

[B4] Atrey A. Clary K. Jensen D. (2019). Exploratory not explanatory: counterfactual analysis of saliency maps for deep reinforcement learning. arXiv[preprint].arXiv:1912.05743. 10.48550/arXiv.1912.05743

[B5] Axmacher N. Henseler M. M. Jensen O. Weinreich I. Elger C. E. Fell J. (2010). Cross-frequency coupling supports multi-item working memory in the human hippocampus. Proc. Natl. Acad. Sci. U.S.A. 107, 3228–3233. 10.1073/pnas.0911531107 20133762 PMC2840289

[B6] Bajcsy R. Aloimonos Y. Tsotsos J. K. (2018). Revisiting active perception. Auton. Robots 42, 177–196. 10.1007/s10514-017-9615-3 31983809 PMC6954017

[B7] Balestrieri E. Ronconi L. Melcher D. (2021). Shared resources between visual attention and visual working memory are allocated through rhythmic sampling. Eur. J. Neurosci. 55, 3040–3053. 10.1111/EJN.15264/v2/response1 33942394

[B8] Begum M. Karray F. (2010). Visual attention for robotic cognition: a survey. IEEE Trans. Auton. Ment. Dev. 3, 92–105. 10.1109/TAMD.2010.2096505

[B9] Benedetto A. Morrone M. C. (2017). Saccadic suppression is embedded within extended oscillatory modulation of sensitivity. J. Neurosci. 37, 3661–3670. 10.1523/JNEUROSCI.2390-16.2016 28270573 PMC6596918

[B10] Benedetto A. Morrone M. C. Tomassini A. (2020). The common rhythm of action and perception. J. Cogn. Neurosci. 32, 187–200. 10.1162/jocn_a_01436 31210564 PMC6938410

[B11] Borji A. Itti L. (2012). State-of-the-art in visual attention modeling. IEEE Trans. Pattern Anal. Mach. Intell. 35, 185–207. 10.1109/TPAMI.2012.89 22487985

[B12] Bos F. Meera A. A. Benders D. Wisse M. (2021). Free energy principle for state and input estimation of a quadcopter flying in wind. arXiv[preprint].arXiv:2109.12052. 10.48550/arXiv.2109.12052

[B13] Brown H. Adams R. A. Parees I. Edwards M. Friston K. (2013). Active inference, sensory attenuation and illusions. Cogn. Process. 14, 411–427. 10.1007/s10339-013-0571-3 23744445 PMC3824582

[B14] Brzezicka A. Kamiński J. Reed C. M. Chung J. M. Mamelak A. N. Rutishauser U. (2019). Working memory load-related theta power decreases in dorsolateral prefrontal cortex predict individual differences in performance. J. Cogn. Neurosci. 31, 1290–1307. 10.1162/jocn_a_01417 31037988 PMC6669115

[B15] Busch N. A. VanRullen R. (2010). Spontaneous eeg oscillations reveal periodic sampling of visual attention. Proc. Natl. Acad. Sci. U.S.A. 107, 16048–16053. 10.1073/pnas.1004801107 20805482 PMC2941320

[B16] Butko N. J. Zhang L. Cottrell G. W. Movellan J. R. (2008). Visual saliency model for robot cameras, in 2008 IEEE International Conference on Robotics and Automation (Pasadena, CA: IEEE), 2398–2403.

[B17] Bylinskii Z. Judd T. Borji A. Itti L. Durand F. Oliva A.. (2019). Mit Saliency Benchmark. Available online at: http://saliency.mit.edu/

[B18] Clark A. (2013). The many faces of precision (replies to commentaries on “whatever next? neural prediction, situated agents, and the future of cognitive science”). Front. Psychol. 4, 270. 10.3389/fpsyg.2013.00270 23734133 PMC3659294

[B19] Crevecoeur F. Kording K. P. (2017). Saccadic suppression as a perceptual consequence of efficient sensorimotor estimation. Elife 6, e25073. 10.7554/eLife.25073 28463113 PMC5449188

[B20] Da Costa L. Parr T. Sajid N. Veselic S. Neacsu V. Friston K. (2020). Active inference on discrete state-spaces: a synthesis. J. Math. Psychol. 99, 102447. 10.1016/j.jmp.2020.102447 33343039 PMC7732703

[B21] Desimone R. (1996). Neural mechanisms for visual memory and their role in attention. Proc. Natl. Acad. Sci. U.S.A. 93, 13494–13499. 10.1073/pnas.93.24.13494 8942962 PMC33636

[B22] Dugué L. Marque P. VanRullen R. (2015). Theta oscillations modulate attentional search performance periodically. J. Cogn. Neurosci. 27, 945–958. 10.1162/jocn_a_00755 25390199

[B23] Dugué L. Roberts M. Carrasco M. (2016). Attention reorients periodically. Curr. Biol. 26, 1595–1601. 10.1016/j.cub.2016.04.046 27265395 PMC4935543

[B24] Eldar E. Cohen J. D. Niv Y. (2013). The effects of neural gain on attention and learning. Nat Neurosci. 16, 1146–1153. 10.1038/nn.3428 23770566 PMC3725201

[B25] Feldman H. Friston K. (2010). Attention, uncertainty, and free-energy. Front. Hum. Neurosci. 4, 215. 10.3389/fnhum.2010.00215 21160551 PMC3001758

[B26] Ferreira J. F. Dias J. (2014). Attentional mechanisms for socially interactive robots-a survey. IEEE Trans. Auton. Ment. Dev. 6, 110–125. 10.1109/TAMD.2014.2303072

[B27] Fiebelkorn I. C. Kastner S. (2019). A rhythmic theory of attention. Trends Cogn. Sci. 23, 87–101. 10.1016/j.tics.2018.11.009 30591373 PMC6343831

[B28] Fiebelkorn I. C. Kastner S. (2020). Functional specialization in the attention network. Annu. Rev. Psychol. 71, 221–249. 10.1146/annurev-psych-010418-103429 31514578 PMC7026883

[B29] Fiebelkorn I. C. Kastner S. (2021). Spike timing in the attention network predicts behavioral outcome prior to target selection. Neuron 109, 177–188. 10.1016/j.neuron.2020.09.039 33098762 PMC7790940

[B30] Fiebelkorn I. C. Pinsk M. A. Kastner S. (2018). A dynamic interplay within the frontoparietal network underlies rhythmic spatial attention. Neuron 99, 842–853. 10.1016/j.neuron.2018.07.038 30138590 PMC6474777

[B31] Fiebelkorn I. C. Pinsk M. A. Kastner S. (2019). The mediodorsal pulvinar coordinates the macaque fronto-parietal network during rhythmic spatial attention. Nat. Commun. 10, 1–15. 10.1038/s41467-018-08151-4 30644391 PMC6333835

[B32] Fine M. S. Minnery B. S. (2009). Visual salience affects performance in a working memory task. J. Neurosci. 29, 8016–8021. 10.1523/JNEUROSCI.5503-08.2009 19553441 PMC6666026

[B33] Frintrop S. Jensfelt P. (2008). Attentional landmarks and active gaze control for visual slam. IEEE Trans. Rob. 24, 1054–1065. 10.1109/TRO.2008.2004977

[B34] Frintrop S. (2006). VOCUS: A Visual Attention System for Object Detection and Goal-Directed Search, Vol. 3899. Berlin: Springer.

[B35] Friston K. (2010). The free-energy principle: a unified brain theory? Nat. Rev. Neurosci. 11, 127–138. 10.1038/nrn2787 20068583

[B36] Friston K. Adams R. Perrinet L. Breakspear M. (2012). Perceptions as hypotheses: saccades as experiments. Front. Psychol. 3, 151. 10.3389/fpsyg.2012.00151 22654776 PMC3361132

[B37] Friston K. Da Costa L. Hafner D. Hesp C. Parr T. (2021). Sophisticated inference. Neural Comput. 33, 713–763. 10.1162/neco_a_01351 33626312

[B38] Friston K. FitzGerald T. Rigoli F. Schwartenbeck P. Pezzulo G. (2017a). Active inference: a process theory. Neural Comput. 29, 1–49. 10.1162/NECO_a_00912 27870614

[B39] Friston K. Mattout J. Kilner J. (2011). Action understanding and active inference. Biol. Cybern. 104, 137–160. 10.1007/s00422-011-0424-z 21327826 PMC3491875

[B40] Friston K. Rigoli F. Ognibene D. Mathys C. Fitzgerald T. Pezzulo G. (2015). Active inference and epistemic value. Cogn. Neurosci. 6, 187–214. 10.1080/17588928.2015.1020053 25689102

[B41] Friston K. Stephan K. Li B. Daunizeau J. (2010). Generalised filtering. Math. Problems Eng. 2010, 621670. 10.1155/2010/621670

[B42] Friston K. J. Harrison L. Penny W. (2003). Dynamic causal modelling. Neuroimage 19, 1273–1302. 10.1016/S1053-8119(03)00202-7 12948688

[B43] Friston K. J. Lin M. Frith C. D. Pezzulo G. Hobson J. A. Ondobaka S. (2017b). Active inference, curiosity and insight. Neural Comput. 29, 2633–2683. 10.1162/neco_a_00999 28777724

[B44] Friston K. J. Parr T. Yufik Y. Sajid N. Price C. J. Holmes E. (2020). Generative models, linguistic communication and active inference. Neurosci. Biobehav. Rev. 118, 42–64. 10.1016/j.neubiorev.2020.07.005 32687883 PMC7758713

[B45] Friston K. J. Trujillo-Barreto N. Daunizeau J. (2008). Dem: a variational treatment of dynamic systems. Neuroimage 41, 849–885. 10.1016/j.neuroimage.2008.02.054 18434205

[B46] Gazzaley A. Nobre A. C. (2012). Top-down modulation: bridging selective attention and working memory. Trends Cogn. Sci. 16, 129–135. 10.1016/j.tics.2011.11.014 22209601 PMC3510782

[B47] Heeger D. J. (1992). Normalization of cell responses in cat striate cortex. Vis. Neurosci. 9, 181–197. 10.1017/S0952523800009640 1504027

[B48] Helfrich R. F. Fiebelkorn I. C. Szczepanski S. M. Lin J. J. Parvizi J. Knight R. T.. (2018). Neural mechanisms of sustained attention are rhythmic. Neuron 99, 854–865. 10.1016/j.neuron.2018.07.032 30138591 PMC6286091

[B49] Helmholtz H. V. (1925). Treatise on Physiological Optics. Rochester, NY: Optical Society of America.

[B50] Hitz G. Galceran E. Garneau M.-,È. Pomerleau F. Siegwart R. (2017). Adaptive continuous-space informative path planning for online environmental monitoring. J. Field Rob. 34, 1427–1449. 10.1002/rob.21722

[B51] Hogendoorn H. (2016). Voluntary saccadic eye movements ride the attentional rhythm. J. Cogn. Neurosci. 28, 1625–1635. 10.1162/jocn_a_00986 27243615

[B52] Hsieh L.-T. Ranganath C. (2014). Frontal midline theta oscillations during working memory maintenance and episodic encoding and retrieval. Neuroimage 85, 721–729. 10.1016/j.neuroimage.2013.08.003 23933041 PMC3859771

[B53] Itti L. Baldi P. (2009). Bayesian surprise attracts human attention. Vis. Res. 49, 1295–1306. 10.1016/j.visres.2008.09.007 18834898 PMC2782645

[B54] Itti L. Koch C. (2001). Computational modelling of visual attention. Nat. Rev. Neurosci. 2, 194–203. 10.1038/35058500 11256080

[B55] Kanai R. Komura Y. Shipp S. Friston K. (2015). Cerebral hierarchies: predictive processing, precision and the pulvinar. Philos. Trans. R. Soc. B Biol. Sci. 370, 20140169. 10.1098/rstb.2014.0169 25823866 PMC4387510

[B56] Kanwisher N. Wojciulik E. (2000). Visual attention: insights from brain imaging. Nat. Rev. Neurosci. 1, 91–100. 10.1038/35039043 11252779

[B57] Kaplan F. Hafner V. V. (2006). The challenges of joint attention. Interact. Stud. 7, 135–169. 10.1075/is.7.2.04kap

[B58] Kim A. Eustice R. M. (2013). Real-time visual slam for autonomous underwater hull inspection using visual saliency. IEEE Trans. Rob. 29, 719–733. 10.1109/TRO.2012.2235699

[B59] Klein R. M. (2000). Inhibition of return. Trends Cogn. Sci. 4, 138–147. 10.1016/S1364-6613(00)01452-2 10740278

[B60] Klink P. C. Jentgens P. Lorteije J. A. (2014). Priority maps explain the roles of value, attention, and salience in goal-oriented behavior. J. Neurosci. 34, 13867–13869. 10.1523/JNEUROSCI.3249-14.2014 25319682 PMC6705291

[B61] Knudsen E. I. (2007). Fundamental components of attention. Annu. Rev. Neurosci. 30, 57–78. 10.1146/annurev.neuro.30.051606.094256 17417935

[B62] Köster M. Finger H. Graetz S. Kater M. Gruber T. (2018). Theta-gamma coupling binds visual perceptual features in an associative memory task. Sci. Rep. 8, 1–9. 10.1038/s41598-018-35812-7 30523336 PMC6283876

[B63] Kragic D. Björkman M. Christensen H. I. Eklundh J.-O. (2005). Vision for robotic object manipulation in domestic settings. Rob. Auton. Syst. 52, 85–100. 10.1016/j.robot.2005.03.01130959920

[B64] Lanillos P. (2013). Minimum time search of moving targets in uncertain environments (Ph.D. thesis).

[B65] Lanillos P. Cheng G. (2018b). Adaptive robot body learning and estimation through predictive coding, in 2018 IEEE/RSJ International Conference on Intelligent Robots and Systems (IROS) (Madrid: IEEE), 4083–4090.

[B66] Lanillos P. Dean-Leon E. Cheng G. (2016). Yielding self-perception in robots through sensorimotor contingencies. IEEE Trans. Cogn. Dev. Syst. 9, 100–112. 10.1109/TCDS.2016.2627820

[B67] Lanillos P. Ferreira J. F. Dias J. (2015a). Designing an artificial attention system for social robots, in 2015 IEEE/RSJ International Conference on Intelligent Robots and Systems (IROS) (Hamburg: IEEE), 4171–4178.

[B68] Lanillos P. Ferreira J. F. Dias J. (2015b). Multisensory 3D saliency for artificial attention systems, in Proc. 3rd Workshop Recogn (Action Scene Understanding), 1–6.

[B69] Lanillos P. Gan S. K. Besada-Portas E. Pajares G. Sukkarieh S. (2014). Multi-uav target search using decentralized gradient-based negotiation with expected observation. Inf. Sci. 282, 92–110. 10.1016/j.ins.2014.05.054

[B70] Lanillos P. Meo C. Pezzato C. Meera A. A. Baioumy M. Ohata W.. (2021). Active inference in robotics and artificial agents: Survey and challenges. arXiv[preprint].arXiv:2112.01871. 10.48550/arXiv.2112.01871

[B71] Lanillos P. Cheng G. (2018a). Active attention applications in robotics, in International Workshop on Active Vision, Attention, and Learning (Tokyo: IEEE Developmental Learning and Epigenetic Robotics (ICDL-Epirob)).

[B72] LaValle S. M. (2006). Planning Algorithms. Cambridge: Cambridge University Press.

[B73] Lengyel M. Yang S. C.-H. Wolpert D. M. (2016). Active sensing in the categorization of visual patterns. eLife 5, e12215. 10.7554/eLife.12215 26880546 PMC4764587

[B74] Lindley D. V. (1956). On a measure of the information provided by an experiment. Ann. Math. Stat. 27, 986–1005. 10.1214/aoms/1177728069

[B75] Louie K. Glimcher P. W. (2019). Normalization principles in computational neuroscience, in Oxford Research Encyclopedia of Neuroscience. Available online at: https://oxfordre.com/neuroscience/view/10.1093/acrefore/9780190264086.001.0001/acrefore-9780190264086-e-43 (accessed July 7, 2022).

[B76] Marchant R. Ramos F. (2014). Bayesian optimisation for informative continuous path planning, in 2014 IEEE International Conference on Robotics and Automation (ICRA) (Hong Kong: IEEE), 6136–6143.

[B77] Meera A. A. Popović M. Millane A. Siegwart R. (2019). Obstacle-aware adaptive informative path planning for uav-based target search, in 2019 International Conference on Robotics and Automation (ICRA) (Montreal, QC: IEEE), 718–724.

[B78] Meera A. A. Wisse M. (2020). Free energy principle based state and input observer design for linear systems with colored noise, in 2020 American Control Conference (ACC) (Denver, CO: IEEE), 5052–5058.

[B79] Meera A. A. Wisse M. (2021a). A brain inspired learning algorithm for the perception of a quadrotor in wind. arXiv[preprint].arXiv:2109.11971. 10.48550/arXiv.2109.11971

[B80] Meera A. A. Wisse M. (2021b). On the convergence of dem's linear parameter estimator, in Machine Learning and Principles and Practice of Knowledge Discovery in Databases (Cham: Springer International Publishing), 692–700.

[B81] Meera A. A. Wisse M. (2022). Free energy principle for the noise smoothness estimation of linear systems with colored noise. arXiv[preprint].arXiv:2204.01796. 10.48550/arXiv.2204.01796

[B82] Mirza M. B. Adams R. A. Friston K. Parr T. (2019). Introducing a bayesian model of selective attention based on active inference. Sci. Rep. 9, 1–22. 10.1038/s41598-019-50138-8 31558746 PMC6763492

[B83] Mirza M. B. Adams R. A. Mathys C. D. Friston K. J. (2016). Scene construction, visual foraging, and active inference. Front. Comput. Neurosci. 10, 56. 10.3389/fncom.2016.00056 27378899 PMC4906014

[B84] Morén J. Ude A. Koene A. Cheng G. (2008). Biologically based top-down attention modulation for humanoid interactions. Int. J. Humanoid Rob. 5, 3–24. 10.1142/S0219843608001285

[B85] Mousavi S. Schukat M. Howley E. Borji A. Mozayani N. (2016). Learning to predict where to look in interactive environments using deep recurrent q-learning. arXiv[preprint].arXiv:1612.05753. 10.48550/arXiv.1612.05753

[B86] Nagai Y. Hosoda K. Morita A. Asada M. (2003). A constructive model for the development of joint attention. Conn. Sci. 15, 211–229. 10.1080/09540090310001655101

[B87] Nakajima M. Schmitt L. I. Halassa M. M. (2019). Prefrontal cortex regulates sensory filtering through a basal ganglia-to-thalamus pathway. Neuron 103, 445–458. 10.1016/j.neuron.2019.05.026 31202541 PMC6886709

[B88] Nakayama R. Motoyoshi I. (2019). Attention periodically binds visual features as single events depending on neural oscillations phase-locked to action. J.Neurosci. 39, 4153–4161. 10.1523/JNEUROSCI.2494-18.2019 30886011 PMC6529869

[B89] Oberauer K. (2019). Working memory and attention-a conceptual analysis and review. J. Cogn. 2, 58. 10.5334/joc.58 31517246 PMC6688548

[B90] Oliver G. Lanillos P. Cheng G. (2021). An empirical study of active inference on a humanoid robot. IEEE Trans. Cogn. Dev. Syst. 14, 462–471. 10.1109/TCDS.2021.3049907

[B91] Orabona F. Metta G. Sandini G. (2005). Object-based visual attention: a model for a behaving robot, in 2005 IEEE Computer Society Conference on Computer Vision and Pattern Recognition (CVPR'05)-Workshops (San Diego, CA: IEEE), 89–89.

[B92] Oudeyer P.-Y. Kaplan F. (2009). What is intrinsic motivation? a typology of computational approaches. Front. Neurorob. 1, 6. 10.3389/neuro.12.006.2007 18958277 PMC2533589

[B93] Panichello M. F. Buschman T. J. (2021). Shared mechanisms underlie the control of working memory and attention. Nature 592, 601–605. 10.1038/s41586-021-03390-w 33790467 PMC8223505

[B94] Parr T. Benrimoh D. A. Vincent P. Friston K. J. (2018). Precision and false perceptual inference. Front. Integr. Neurosci. 12, 39. 10.3389/fnint.2018.00039 30294264 PMC6158318

[B95] Parr T. Corcoran A. W. Friston K. J. Hohwy J. (2019). Perceptual awareness and active inference. Neurosci. Consciousness 2019, niz012. 10.1093/nc/niz012 31528360 PMC6734140

[B96] Parr T. Friston K. J. (2017a). Uncertainty, epistemics and active inference. J. R. Soc. Interface 14, 20170376. 10.1098/rsif.2017.0376 29167370 PMC5721148

[B97] Parr T. Friston K. J. (2017b). Working memory, attention, and salience in active inference. Sci. Rep. 7, 1–21. 10.1038/s41598-017-15249-0 29116142 PMC5676961

[B98] Parr T. Friston K. J. (2019). Attention or salience? Curr. Opin. Psychol. 29, 1–5. 10.1016/j.copsyc.2018.10.006 30359960

[B99] Parr T. Pezzulo G. (2021). Understanding, explanation, and active inference. Front. Syst. Neurosci. 15, 772641. 10.3389/fnsys.2021.772641 34803619 PMC8602880

[B100] Parr T. Sajid N. Da Costa L. Mirza M. B. Friston K. J. (2021). Generative models for active vision. Front. Neurorobot. 15, 651432. 10.3389/fnbot.2021.651432 33927605 PMC8076554

[B101] Peters B. Kaiser J. Rahm B. Bledowski C. (2020). Object-based attention prioritizes working memory contents at a theta rhythm. J. Exp. Psychol. Gen. 150, 1250–1256. 10.1037/xge0000994 33211526

[B102] Phillips J. M. Kambi N. A. Saalmann Y. B. (2016). A subcortical pathway for rapid, goal-driven, attentional filtering. Trends Neurosci. 39, 49–51. 10.1016/j.tins.2015.12.003 26743499

[B103] Pomper U. Ansorge U. (2021). Theta-rhythmic oscillation of working memory performance. Psychol. Sci. 32, 1801–1810. 10.1177/09567976211013045 34592108

[B104] Popović M. Vidal-Calleja T. Hitz G. Sa I. Siegwart R. Nieto J. (2017). Multiresolution mapping and informative path planning for uav-based terrain monitoring, in 2017 IEEE/RSJ International Conference on Intelligent Robots and Systems (IROS) (Vancouver, BC: IEEE), 1382–1388.

[B105] Rao R. P. (2005). Bayesian inference and attentional modulation in the visual cortex. Neuroreport 16, 1843–1848. 10.1097/01.wnr.0000183900.92901.fc 16237339

[B106] Rasouli A. Lanillos P. Cheng G. Tsotsos J. K. (2020). Attention-based active visual search for mobile robots. Auton. Robots 44, 131–146. 10.1007/s10514-019-09882-z

[B107] Raybourn M. S. Keller E. L. (1977). Colliculoreticular organization in primate oculomotor system. J. Neurophysiol. 40, 861–878. 10.1152/jn.1977.40.4.861 407334

[B108] Reynolds J. H. Heeger D. J. (2009). The normalization model of attention. Neuron 61, 168–185. 10.1016/j.neuron.2009.01.002 19186161 PMC2752446

[B109] Reynolds J. H. Pasternak T. Desimone R. (2000). Attention increases sensitivity of v4 neurons. Neuron 26, 703–714. 10.1016/S0896-6273(00)81206-4 10896165

[B110] Rizzolatti G. Riggio L. Dascola I. Umiltá C. (1987). Reorienting attention across the horizontal and vertical meridians: evidence in favor of a premotor theory of attention. Neuropsychologia 25, 31–40. 10.1016/0028-3932(87)90041-8 3574648

[B111] Roberts R. Ta D.-N. Straub J. Ok K. Dellaert F. (2012). Saliency detection and model-based tracking: a two part vision system for small robot navigation in forested environment, in Unmanned Systems Technology XIV, Vol. 8387 (International Society for Optics and Photonics), 83870S.

[B112] Rucci M. Ahissar E. Burr D. (2018). Temporal coding of visual space. Trends Cogn. Sci. 22, 883–895. 10.1016/j.tics.2018.07.009 30266148 PMC6179437

[B113] Ruff D. A. Cohen M. R. (2016). Stimulus dependence of correlated variability across cortical areas. J. Neurosci. 36, 7546–7556. 10.1523/JNEUROSCI.0504-16.2016 27413163 PMC4945672

[B114] Sajid N. Da Costa L. Parr T. Friston K. (2021a). Active inference, bayesian optimal design, and expected utility. arXiv[preprint].arXiv:2110.04074. 10.1017/9781009026949.007

[B115] Sajid N. Faccio F. Da Costa L. Parr T. Schmidhuber J. Friston K. (2021b). Bayesian brains and the r\'enyi divergence. arXiv[preprint].arXiv:2107.05438. 10.48550/arXiv.2107.05438 35231935

[B116] Sajid N. Friston K. J. Ekert J. O. Price C. J. Green D. W. (2020). Neuromodulatory control and language recovery in bilingual aphasia: An active inference approach. Behav. Sci. 10, 161. 10.3390/bs10100161 33096824 PMC7588909

[B117] Sajid N. Holmes E. Costa L. D. Price C. Friston K. (2022). A mixed generative model of auditory word repetition. bioRxiv [preprint]. 10.1101/2022.01.20.477138

[B118] Santangelo V. (2015). Forced to remember: when memory is biased by salient information. Behav. Brain Res. 283, 1–10. 10.1016/j.bbr.2015.01.013 25595422

[B119] Santangelo V. Di Francesco S. A. Mastroberardino S. Macaluso E. (2015). Parietal cortex integrates contextual and saliency signals during the encoding of natural scenes in working memory. Hum. Brain Mapp. 36, 5003–5017. 10.1002/hbm.22984 26333392 PMC6869543

[B120] Santangelo V. Macaluso E. (2013). Visual salience improves spatial working memory via enhanced parieto-temporal functional connectivity. J. Neurosci. 33, 4110–4117. 10.1523/JNEUROSCI.4138-12.2013 23447619 PMC3695392

[B121] Schmitz T. W. Duncan J. (2018). Normalization and the cholinergic microcircuit: a unified basis for attention. Trends Cogn. Sci. 22, 422–437. 10.1016/j.tics.2018.02.011 29576464

[B122] Shon A. P. Grimes D. B. Baker C. L. Hoffman M. W. Zhou S. Rao R. P. (2005). Probabilistic gaze imitation and saliency learning in a robotic head, in Proceedings of the 2005 IEEE International Conference on Robotics and Automation (Barcelona: IEEE), 2865–2870.

[B123] Sommer M. A. Wurtz R. H. (2006). Influence of the thalamus on spatial visual processing in frontal cortex. Nature 444, 374–377. 10.1038/nature05279 17093408

[B124] Spratling M. W. (2008). Predictive coding as a model of biased competition in visual attention. Vis. Res. 48, 1391–1408. 10.1016/j.visres.2008.03.009 18442841

[B125] Tomassini A. Ambrogioni L. Medendorp W. P. Maris E. (2017). Theta oscillations locked to intended actions rhythmically modulate perception. Elife 6, e25618. 10.7554/eLife.25618 28686161 PMC5553936

[B126] Treisman A. M. Gelade G. (1980). A feature-integration theory of attention. Cogn. Psychol. 12, 97–136. 10.1016/0010-0285(80)90005-5 7351125

[B127] Tsotsos J. K. Culhane S. M. Wai W. Y. K. Lai Y. Davis N. Nuflo F. (1995). Modeling visual attention via selective tuning. Artif. Intell. 78, 507–545. 10.1016/0004-3702(95)00025-9

[B128] Ude A. Wyart V. Lin L.-H. Cheng G. (2005). Distributed visual attention on a humanoid robot, in 5th IEEE-RAS International Conference on Humanoid Robots, 2005 (Tsukuba: IEEE), 381–386.

[B129] VanRullen R. (2016). Perceptual cycles. Trends Cogn. Sci. 20, 723–735. 10.1016/j.tics.2016.07.006 27567317

[B130] Welch G. Bishop G. (2002). An Introduction to the Kalman Filter. Chapel Hill, NC: University of North Carolina.

[B131] White B. J. Berg D. J. Kan J. Y. Marino R. A. Itti L. Munoz D. P. (2017). Superior colliculus neurons encode a visual saliency map during free viewing of natural dynamic video. Nat. Commun. 8, 1–9. 10.1038/ncomms14263 28117340 PMC5286207

[B132] Whiteley L. Sahani M. (2008). Implicit knowledge of visual uncertainty guides decisions with asymmetric outcomes. J. Vis. 8, 2–2. 10.1167/8.3.2 18484808 PMC2515365

[B133] Yang S. C.-H. Lengyel M. Wolpert D. M. (2016a). Active sensing in the categorization of visual patterns. Elife 5, e12215. 10.7554/eLife.12215 26880546 PMC4764587

[B134] Yang S. C.-H. Wolpert D. M. Lengyel M. (2016b). Theoretical perspectives on active sensing. Curr. Opin. Behav. Sci. 11, 100–108. 10.1016/j.cobeha.2016.06.009 30175197 PMC6116896

